# The Role of Genetic, Environmental, and Dietary Factors in Alzheimer’s Disease: A Narrative Review

**DOI:** 10.3390/ijms26031222

**Published:** 2025-01-30

**Authors:** Beyza Mertaş, İ. İpek Boşgelmez

**Affiliations:** 1 Department of Pharmacology, Faculty of Pharmacy, Düzce University, Düzce 81010, Türkiye; beyzamertas@duzce.edu.tr; 2 Department of Toxicology, Faculty of Pharmacy, Erciyes University, Kayseri 38280, Türkiye

**Keywords:** Alzheimer’s disease, risk factors, exposome, genetics, gut microbiota, infections, diet

## Abstract

Alzheimer’s disease (AD) is one of the most common and severe forms of dementia and neurodegenerative disease. As life expectancy increases in line with developments in medicine, the elderly population is projected to increase in the next few decades; therefore, an increase in the prevalence of some diseases, such as AD, is also expected. As a result, until a radical treatment becomes available, AD is expected to be more frequently recorded as one of the top causes of death worldwide. Given the current lack of a cure for AD, and the only treatments available being ones that alleviate major symptoms, the identification of contributing factors that influence disease incidence is crucial. In this context, genetic and/or epigenetic factors, mainly environmental, disease-related, dietary, or combinations/interactions of these factors, are assessed. In this review, we conducted a literature search focusing on environmental factors such as air pollution, toxic elements, pesticides, and infectious agents, as well as dietary factors including various diets, vitamin D deficiency, social factors (e.g., tobacco and alcohol use), and variables that are affected by both environmental and genetic factors, such as dietary behavior and gut microbiota. We also evaluated studies on the beneficial effects of antibiotics and diets, such as the Mediterranean-DASH Intervention for Neurodegenerative Delay (MIND) and Mediterranean diets.

## 1. Introduction

Alzheimer’s disease (AD) constitutes the most prevalent form of dementia, accounting for 60–70% of diagnosed cases [1]. The World Health Organization (WHO) defines dementia as an umbrella term for several diseases that progressively affect memory, other cognitive functions, and behavior, resulting in significant interference with a person’s ability to carry out daily activities. Compared to 57.4 million individuals living with dementia worldwide in 2019 [2], the projections for 2030, 2040, and 2050 have been estimated to increase to 83.2 million, 116.0 million, and 152.8 million, respectively [2,3]. Approximately 9.9 million individuals worldwide develop dementia each year, equating to a new case every three seconds. This staggering statistic emphasizes the critical need for focused and strengthened efforts in this field. Nearly 60% of individuals with dementia reside in low- and middle-income countries, where most new cases (71%) are expected to occur. Dementia is among the major causes of disability and functional dependency in the elderly and is currently the seventh highest cause of death globally [3]. In the United States, official death records showed 121,499 deaths from AD in 2019, making it the sixth highest cause of death [4]. According to official death statistics in Türkiye, the number of older adults who lost their lives due to AD was 13,859 in 2018, representing the highest level in the last decade, while the data in 2022 showed a slight fall in number to 11,880 (mean 4.6% vs. 3.2%) [5].

**Figure 1:**
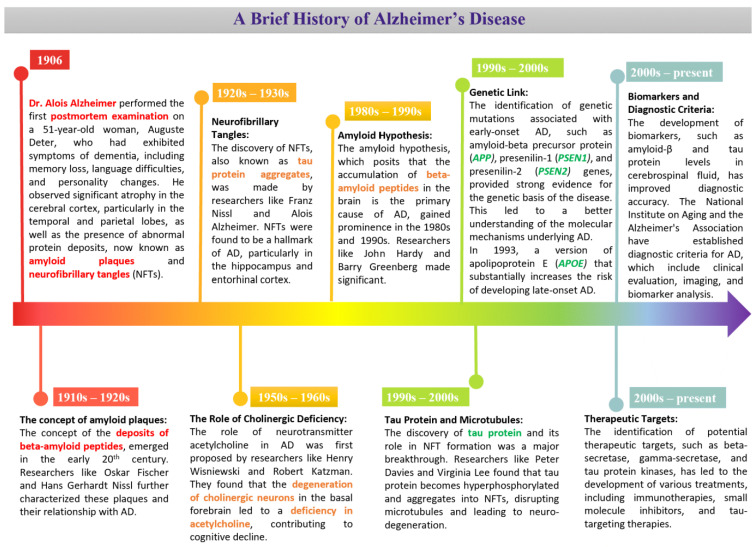
A brief history of Alzheimer’s disease [6,7].

**Figure 2:**
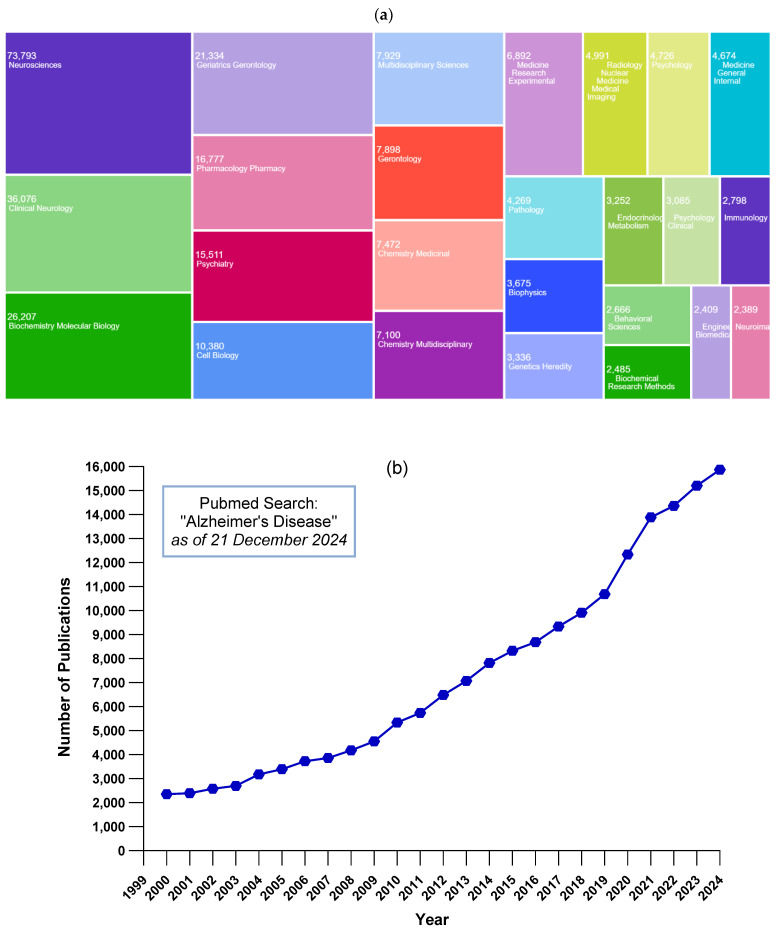
(**a**) Distribution of the top 25 “Web of Science Categories” within the published items related to the term “Alzheimer’s disease” in the tree map, as of 21 December 2024. The distribution of “Web of Science Categories” within the published items on this topic. (**b**) The number of publications in “Web of Science” between 2000 and 2024 per year related to the search term “Alzheimer’s disease”.

The critical factors in AD can be traced back to Alois Alzheimer’s initial observations and have since been refined through decades of research [6,7] (Figure 1). Today, we have gained a clearer understanding of the molecular mechanisms, genetic links, and diagnostic criteria, paving the way for novel treatment development. Studies into AD focus on different areas of expertise, including but not limited to neuroscience, clinical neurology, biochemistry/molecular biology, geriatrics/gerontology, pharmacology/pharmacy, psychiatry, cell biology, multidisciplinary sciences, and medicinal chemistry (as depicted in Figure 2a). From 1995 to 2018, the number of publications in the field increased gradually (Figure 2b) and an upsurge in AD-related papers has been observed after 2018, which may be a result of various factors, such as the Coronavirus disease 2019 (COVID-19) pandemic. The numbers reached the highest recorded level, and showed a stable upward trend, in the 2021–2024 period, and a similar trend for 2025 is likely to follow. As of 21 December 2024, search terms resulted in 15,875 hits for publications in 2024.

The most common symptoms of AD include progressive impairment or decline in memory and cognitive function, changes in behavior leading to aggression, and impaired social skills through different stages. The changes in the brains of AD patients begin in the hippocampus and subsequently affect the entire brain as dementia progresses, resulting in neurodegeneration or neuronal loss. Diagnosis mainly relies on (1) neuroimaging markers obtained through the detection of amyloid and neurofibrillary tangles (NFT) caused by the aggregation of hyperphosphorylated tau proteins [8] in positron emission tomography (PET) scans or cerebrospinal fluid (CSF) analysis, and (2) blood biomarkers such as amyloid β peptides (Aβ42-to-Aβ40 ratio) and phosphorylated tau (p-tau) isoforms (e.g., p-tau217, p-tau181, p-tau231, and p-tau205) [9]. Some other blood biomarkers, such as brain-derived total tau proteins and β-synuclein, are also available.

A recent study by Zeng et al. demonstrated that microglia are the initial responders to Aβ by aggregating around amyloid plaques [10]. Furthermore, this study showed a core shell structure comprising oligodendrocyte precursor cells (OPCs) and disease-associated astrocyte-like (DAA-like) cells in outer shells. In contrast, Aβ plaques and disease-associated microglia (DAM) were found in inner shells. Additionally, hyperphosphorylated tau was found to be associated with oligodendrocyte subtypes but not with Aβ plaques. The researchers highlighted the uncertainty surrounding the potential benefits and harms of oligodendrocyte subtype elevation, noting that it could either support axons damaged by tauopathy, repair damaged myelin, or worsen tauopathy [10]. Recently, Baligács et al. proposed “a dual-phase response” of microglia in AD. Their study illustrated how non-reactive microglia contribute to the initiation of evident amyloid plaques, with a decrease in plaque counts and neuritic dystrophy when microglia were depleted prior to the deposition of Aβ plaques. Moreover, transplantation of human microglia at this stage led to a revival in plaque development. In contrast, during later stages, activated microglia played a role in compacting these plaques, thereby limiting their toxicity, implying a protective function, as indicated by the finding that depletion of microglia at this late stage resulted in insufficient plaque compaction and increased neuritic dystrophy [11].

Early diagnosis of AD is the best way to prevent or slow down the course of the disease. AD can be diagnosed at an early stage based on MRI results indicating atrophy of the hippocampus, amygdala, and entorhinal cortex, as well as notable amnesia, which can be confirmed through appropriate tests. Furthermore, changes in CSF biomarkers can be observed, including an increase in tau and/or phosphorylated tau proteins and a decrease in Aβ(1-42) peptides. In addition, PET neuroimaging results, typically seen in AD patients, including decreased glucose metabolism, can also be used to support the diagnosis. The National Institute of Neurological and Communicative Disorders and Stroke/Alzheimer’s Disease and Related Disorder Association (NINCDS-ADRDA) criteria and the Diagnostic and Statistical Manual of Mental Disorders, Fifth Edition (DSM-V) can be employed for diagnostic purposes. The NINCDS-ADRDA criteria are contingent on histological verification and clinical symptoms, with the potential for patients to be classified as definitively diagnosed with AD or as possible or probable AD diagnoses [12]. Revised criteria for diagnosis and staging [13] and a draft guidance [14] for clinical studies are also available (Table 1). Non-invasive, specific, novel biomarkers for early diagnosis or monitoring the progression of AD and potential therapeutic targets, such as cell-free DNA methylation [15], microRNAs (miRNAs) [16], and proteomics- and metabolomics-based panels [17], are evaluated.

Although, in recent years, significant advances in our knowledge of the pathogenesis of AD have paved the way for novel therapeutic options, the search for radical treatment is still ongoing. Available medicines, including anticholinergic drugs and N-methyl-D-aspartate (NMDA) antagonists, help improve the patients’ status or slow down the progression of symptoms. Therefore, drug research and development studies are warranted. Furthermore, in view of the risk factors, several options for prophylaxis, including non-steroidal anti-inflammatory drugs (NSAIDs) and antioxidants, may be of use [8].

**Table 1:** A six-stage numeric clinical staging scheme is available [13] and clinical staging of AD applies only to individuals who are in the AD pathophysiologic continuum. The six clinically defined stages, which may also be of use for the design and evaluation of clinical trials, are as follows: [13,14].

**1**	**Stage 1:** Asymptomatic individuals with biomarker evidence of AD. Subjective complaint, functional impairment, or detectable abnormalities on sensitive neuropsychological measures are absent. Characteristic pathophysiological signs of AD may be demonstrated by assessment of various biomarkers but no evidence of clinical impact.
**2**	**Stage 2:** A transitional stage which denotes the earliest detectable clinical symptoms that might be due to AD in individuals who are cognitively unimpaired. Patients have characteristic pathophysiological changes in AD and subtle detectable abnormalities on sensitive neuropsychological measures or subjective complaints of mild cognitive symptoms but have no functional impairment yet.
**3**	**Stage 3:** These patients have characteristic pathophysiological indicators of AD. More apparent detectable abnormalities on sensitive neuropsychological measures and mild but detectable cognitive impairment resulting in significant functional loss are observed (i.e., inefficient in activities of daily living but still independent). The functional impairment corresponds with the syndrome of “mild cognitive impairment” that may also encompass patients in “late Stage 2” or “early Stage 4”.
**4**–**6**	**Stages 4, 5, and 6:** Patients present with “overt dementia”, progressing through mild, moderate, and severe stages. Loss of independence with progressively worse functional loss is expected.

## 2. Risk Factors

**Figure 3:**
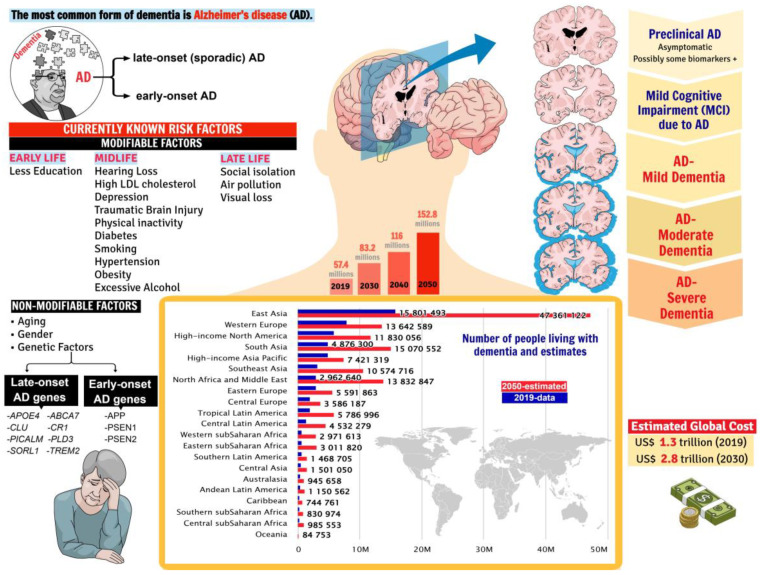
Global prevalence, currently known risk factors, and staging of Alzheimer’s disease [2,13,18]. The genes associated with AD include apolipoprotein E4 (*APOE4*)*,* clusterin (*CLU*), phosphatidylinositol binding clathrin assembly protein (*PICALM*), sortilin-related receptor (*SORL1*), ATP binding cassette subfamily A member 7 (*ABCA7*), complement receptor type 1 (*CR1*), phospholipase D3 (*PLD3*), triggering receptor expressed on myeloid cells 2 (*TREM2*), amyloid-beta precursor protein (*APP*), presenilin-1 (*PSEN1*), and presenilin-2 (*PSEN2*).

Various risk factors of AD have been identified, including nonmodifiable risk factors such as genetics and aging, as well as modifiable risk factors such as environmental pollution or diet, which we can intervene in to implement preventive measures. In this context, a group of specialized research experts identified and grouped 14 factors that may contribute to increased dementia risk according to the stage of life as follows (Figure 3): risk factors in early life (less education), midlife (hearing loss, high LDL cholesterol, depression, traumatic brain injury, physical inactivity, diabetes, smoking, hypertension, obesity, and excessive alcohol), and late-life (social isolation, air pollution, and visual loss) [18]. Overall, modifying these 14 risk factors might prevent or delay up to 45% of dementias.

It is essential to acknowledge that, except for single-gene diseases, most diseases arise from intricate interactions among genetic, environmental, and other risk factors. While single markers can offer insights, they may not provide a conclusive link between environmental exposure and disease. Thus, a more promising approach could involve combining genetic and biological markers to better understand the critical points in the exposure–disease continuum and the methods for deriving these markers [19]. In the pursuit of enhancing health outcomes, various interventions merit consideration, including adopting a healthy diet and using nutraceuticals that target key factors such as oxidative stress, inflammation, and mitochondrial dynamics. These strategies also focus on the interaction between mitochondria and disease-related proteins. Furthermore, the interplay between numerous factors, including mitochondrial activity and disease-related proteins, deserves careful consideration. It is also paramount to assess the potential impacts of environmental contaminants, such as air pollution and prolonged exposure to pesticides, as these factors can play a significant role [20].

An important point appears to be relationship between education and the *APOE ɛ4* variant gene. Interestingly, expected cognitive decline caused by *APOE* ε4 may be moderated by advanced education [21,22]. In this vein, it has been suggested that *APOE* ε4 carriers may benefit from at least 16 years of schooling as a protective strategy against the adverse cognitive effects related to this specific gene [21]. Regarding young-onset dementia, in a cohort study involving 356,052 participants from the UK Biobank, a variety of modifiable and nonmodifiable risk factors were evaluated. Factors related to a reduced incidence risk included higher levels of formal education, lower physical frailty (indicated by greater handgrip strength), and moderate alcohol consumption (compared to abstinence), whereas increased risk was found to be related to low socioeconomic status, having two *APOE* ε4 alleles, alcohol use disorder, social isolation, vitamin D deficiency, elevated C-reactive protein levels, hearing impairment, orthostatic hypotension, stroke, diabetes, heart disease, and depression [23].

### 2.1. Aging

A well-known risk factor of AD is aging. Various mechanisms, including higher levels of growth factors, efficient energy metabolism, clearance of misfolded proteins, and other regulatory processes, may protect the young brain against AD; however, a decline in these protective mechanisms either as a result of aging-related or other factors may contribute to the development of AD [24]. However, we cannot attribute AD only as an “aging-linked” disease or as an inevitable consequence of aging [25]; thus, the effects of dementia on younger individuals should not be overlooked. A recent report by Li et al. extracted data from the 2021 Global Burden of Diseases, Injuries, and Risk Factors Study to assess dementia status in younger individuals. Their findings indicated that over the last three decades, the global prevalence, incidence, mortality, and disability-adjusted life years (DALY) associated with dementia in people under 70 years of age have doubled. Additionally, there has been a notable acceleration in the growth rate of young-onset dementia cases over the last decade [26]. The authors underlined that both globally and regionally, females aged <70 years consistently exhibited higher incidence, mortality, and DALY rates for dementia than males. Of note, a common aspect of aging is impaired sleep, which may be related to the onset of AD. Results of a recent study on a murine model of AD (*APP/PS1*) and young wild-type mice comparing the effects of 7 hours of sleep deprivation showed that the inability of *APP/PS1* mice to enhance norepinephrine oscillations following sleep deprivation may contribute to glymphatic dysfunction, increased vulnerability to sleep loss, and subsequent Aβ buildup, which is also supported by proteome analysis showing altered protein clearance [27].

In the realm of neuroplasticity, the Red Queen hypothesis merits academic consideration [28]. This hypothesis, first coined by Van Valen [29], derives its name from a statement by the Red Queen, a fictional character in Lewis Carroll’s “Through the Looking Glass” in which she states, “Now, here, you see, it takes all the running you can do, to keep in the same place. If you want to get somewhere else, you must run at least twice as fast as that!” In this sense, the Red Queen hypothesis can be articulated as continuous and dynamic adaptation and evolution to keep pace with the pressure exerted by rapidly evolving competitive conditions or stresses. In relation to neuroplasticity, this hypothesis emphasizes that the competition for available plasticity and the compensatory mechanisms that counteract neurodegeneration have a detrimental impact on the formation of memory traces. Throughout the aging process, the mechanisms facilitating neuroplasticity must function at their maximum capacity to preserve memory traces within the brain. This necessity increases the risk of failure in neuroplasticity, alongside the challenge of utilizing the remaining neuronal plasticity to either acquire new information or to restore and preserve neuronal circuits. To prevent, retard, or mitigate cognitive decline associated with aging, one potential approach is to enter the later stages of life with the highest attainable level of neuronal plasticity and to continuously endeavor to sustain this state [28].

### 2.2. Gender

Research indicates that women demonstrate a greater prevalence of AD, underscoring the critical need for focused efforts to enhance our understanding of this issue. Nichols et al. [2] reported that the female-to-male ratio among individuals with dementia in 2019 was 1.69 (1.64–1.73), and they predict that this pattern will continue until 2050 (1.67 [1.52–1.85]). Gender differences in AD can partly be attributed to women having a higher life expectancy than men; however, the underlying mechanisms of AD may also play a role [30]. Several possible reasons for the variability in the prevalence of AD dementia between genders include, but are not limited to, the observation that the risks associated with the *APOE4* allele appear to be more pronounced in women. Moreover, there are distinctions between genders in how the shortening of telomeres responds to aging, neurodegenerative processes, and hormonal fluctuations. In particular, women who are at an elevated risk of AD may experience a phase in which increased estrogen levels enhance cognitive function. Furthermore, complications during pregnancy, such as gestational hypertension and preeclampsia, may be associated with a heightened risk of developing dementia in the future. Variations in education may contribute to disparities in cognitive reserve. Additionally, gender differences in psychiatric co-morbidities, particularly the higher prevalence of depression and insomnia in women, could increase the risk of AD. These factors may play a significant role in this context [31]. A comprehensive cohort study involving 1.6 million middle-aged women has revealed that participants diagnosed with depression have a significantly elevated risk of developing young-onset dementia compared to their counterparts without depression. Specifically, premenopausal women with depression demonstrated a 2.67-fold increase in risk, while postmenopausal women exhibited a 2.5-fold increase when compared to those without depression. Additionally, for premenopausal women who did not experience depression, late menarche (onset after the age of 16) correlates with a 1.5-fold increase in the risk of young-onset dementia compared to the reference group. In postmenopausal women, an earlier onset of menopause has been associated with a higher risk [32]. The most comprehensive genetic analysis examining late-life memory performance, which accounted for gender-specific and cross-ancestral factors, focused on identifying candidate genes and pathways associated with memory and highlighted three sex-specific loci: rs67099044—*CBLN2*, rs719070—*SCHIP1/IQCJ-SCHIP*, and rs5935633—*EGL6/TCEANC/OFD1*. Remarkably, the latter locus is located on the X chromosome and is associated with memory decline in females [33]. The findings suggest that the heritable aspects of late-life memory appear to be comparable between women and men. Furthermore, researchers identified a correlation between heparan sulfate signaling pathways— associated with the neuropathological progression of AD and disorders that predominantly affect females—and baseline memory performance within a cross-ancestry cohort of women. This study proposed that the upregulation of heparan signaling pathways may be associated with improved memory performance among females. As a result, researchers have highlighted the significance of understanding the sex-specific genetic components that influence memory. Identifying these genes and pathways and their link to AD is crucial, as they may represent potential targets for future therapeutic approaches. A recent observational study suggested that hormone replacement therapy (HRT) is associated with lower tau neuroimaging and fluid biomarkers in postmenopausal females [34] and has also been shown to be beneficial in at-risk *APOE4* women [35]. However, a cross-sectional study showed an elevated tau PET signal among postmenopausal women with late HRT initiation compared to those who started HRT before menopause onset. In addition, another factor related to the elevated tau PET is HRT treatment among those with high levels of Aβ [36]. The results are consistent with the critical window hypothesis, which posits that the impact of HRT relies on the timing of its commencement relative to age and/or the onset of menopause. The hypothesis suggests that the benefits of HRT are contingent upon its early initiation [37,38,39]. Overall, while the literature demonstrates a promising effect of HRT on AD, the beneficial or detrimental effect of this therapy may depend on several factors, such as the patient’s age at HRT initiation and baseline characteristics, including genotype and cardiovascular health, as well as the dosage, formulation, and duration of HRT [40].

### 2.3. Genetic Factors

The role of genetics is a matter of concern, especially for the family members of AD patients. Several genetic variants have been suggested to be associated with AD, and these may affect the risk of developing the disease; however, in most cases, AD is influenced by multiple genes in combination with lifestyle and environmental factors (Figure 3). Regarding the genetic factors, if the individual carries more than one genetic variant or group of variants that can increase the risk of AD or has a parent and/or a sibling diagnosed with AD, the risk may be higher than for a person without the factors mentioned above. It should be kept in mind that people who develop AD do not always have a history of the disease in their families [41]. Understanding which genes play a role—and what role they play—may help identify new methods to prevent, delay, or treat dementia. Discovery of all the related genes and their roles in the pathology of AD is of particular importance since the pharmacological agents that aim to change the course of the disease are currently sparse [42] and, due to genetic factors such as the *APOE4* allele, some adverse reactions may hamper the use of available treatment options. Certain genetic mutations in some other genes, such as *APP*, *PSEN1*, and *PSEN2*, may also increase the risk of developing AD. A parent who carries one of these genes has a 50% probability of transferring it to their children, thus significantly increasing the children’s risk of developing early (young)-onset AD [41]. The *APOE ε4* allele is the most significant genetic risk factor for late-onset AD and is associated with an increased risk of developing the disease [41]. In vivo studies showed that *APOE4* caused a significant increase in both Aβ accumulation and plaques in mice, as well as in the formation and aggregation of new plaques [42,43,44]. Moreover, *APOE4*-expressing neurons have tau pathogenesis, neuroinflammation, and tau-mediated neurodegeneration, independently of Aβ pathology [45]. Recently, researchers proposed that *APOE4* homozygotes should also be considered as a different form of genetically determined AD, as Autosomal-dominant Alzheimer’s disease (ADAD) and Down syndrome-associated Alzheimer’s disease [46,47]. Regarding late-onset AD, some other genes, such as complement receptor type 1 (*CR1*), clusterin (*CLU*), phospholipase D3 (*PLD3*), and ATP binding cassette subfamily A member 7 (*ABCA7*) are known. Even though further studies are needed, the *ABCA7* gene is thought to be related to Aβ production and deposition [48]. Likewise, the *CR1* gene is another gene regulating Aβ pathology. This gene is present in microglia and takes part in removing Aβ in the AD brain [49,50]. *PLD3* has also been linked to Aβ regulation and is overexpressed in the brain, a decline in its expression has been observed in the brains of individuals with AD, and represents a promising new target for therapeutic intervention [51]. *CLU* is the third most significant genetic risk factor for late-onset AD. A substantial number of studies have demonstrated that the levels of neuronal *CLU* are elevated in terms of inflammation, AD, and injury. However, whether this increase is beneficial or harmful remains a topic of contention [52,53,54].

In a genome-wide association analysis comprising 3046 participants from 12 different studies, Nho et al. have found novel genetic variants in *cytochrome P450 1B1 (CYP1B1)-RMDN2* locus that affect medial temporal lobe and cortical tau levels measured by PET, shedding light on the genetic underpinnings of cerebral tau deposition and supporting novel pathways for therapeutic developments in AD [55]. The findings underscored the association of tau deposition and accelerated cognitive decline with *CYP1B1-RMDN2* locus, with the strongest signal, located at rs2113389, explaining 4.3% of the variation in cortical tau, whereas *APOE4* rs429358 explains 3.6%. While additive effects were shown between rs2113389 and Aβ positivity, *APOE4*, and diagnosis, no interactions were evident. AD is associated with increased expression of *CYP1B1*, and *rs2113389* is associated with increased *CYP1B1* expression and methylation levels. Mouse model studies provide further functional evidence for a link between *CYP1B1* and tau deposition but not Aβ. Recently, Zhou et al. conducted a logistic regression analysis that revealed sortilin-related receptor (*SORL1*) haplotypes associated with AD in East Asian (N = 5249) and European (N = 8588) populations. Their research uncovered an isoform-specific missense variant in haplotype Hap_A (rs2282647-C allele), which modifies the function and levels of a truncated SORL1 protein isoform that has not been thoroughly studied. The authors noted that the SORL1 haplotype Hap_A, prevalent in East Asian populations, correlated with cognitive abilities, brain volume, and the activity of specific neuronal and immune-related pathways closely linked to AD risk. Furthermore, it was associated with reduced expression of the truncated SORL1 protein isoform, indicating possible mechanisms for the protective effects against SORL1 in AD, both for *APOE ε4* carriers and non-carriers [56]. A recent study involving 800,597 participants identified 13 novel suggestive risk loci associated with all-cause dementia. Among the loci examined, one was found near the semaphorin-4D (*SEMA4D*) gene, which plays a regulatory role in various processes related to neuroinflammation and neurodegeneration, including triggering the activation of inflammatory microglia [57]. Targeting *SEMA4D* with antibody blockade presents a potential disease-modifying strategy to slow cognitive decline in early-stage Huntington’s disease, and it may also offer valuable benefits for individuals with all-cause dementia [58]. The other significant genetic loci have been identified in the zinc finger protein 652 (*ZNF652*) gene, linked to various effects including the risk of hypertension, and also near the anoctamin-3 (*ANO3*) gene, responsible for encoding the transmembrane protein anoctamin-3 and associated with focal dystonia [57]. Further exploration of the genetic risk factors associated with AD is warranted, holding out hope for the potential to deepen our understanding of the mechanisms and pathways involved in the disease. Furthermore, these investigations could help identify new therapeutic targets.

### 2.4. Diseases

Hypertension, obesity, depression, diabetes, hearing loss, and traumatic brain injury are currently known midlife risk factors of AD, while visual loss is cited as late-life risk factor [18]. Table 2 outlines the diseases and their corresponding mechanisms associated with AD that have been identified as potential risk factors contributing to the onset and progression of the condition.

A recent meta-analysis highlighted antihypertensive treatment’s beneficial role in AD risk mitigation, especially in late-life [64]. The analyses on 14 nations with 31,250 participants with untreated hypertension showed 36% and 42% increased risk of AD as compared with healthy control and treated hypertension groups, respectively [64]. Another study indicated that the adverse outcomes of hypertension were mainly associated with the presence of white matter hyperintensities in AD patients exhibiting a high Aβ load. In AD patients with a low Aβ load, hypertension has been found to correlate with disease pathology, the thickness of the entorhinal cortex, and the burden of white matter hyperintensities [59]. A recent investigation revealed that obesity in the early stages of AD development exacerbates dysfunction in memory reliant on the dorsal hippocampus (dHC) and disrupts mitochondrial and lipid metabolism in this region. Furthermore, obesity interferes with noradrenergic transmission, neuronal function, and vascular integrity. Another key discovery from the study is that while pathways such as neurotransmission, angiogenesis, metal ion binding, and apoptosis were notably affected in female obese rats on a Western diet, male rats had significant effects related to oxidative phosphorylation.

Additionally, the researchers noted that female rats exhibited considerably higher levels of soluble Aβ1-42 in the dHC than their male counterparts [60]. This research is consistent with the “AD exposome” as it assesses AD alongside various endogenous risk factors such as gender and external/behavioral risk factors such as diet and lifestyle, serving as a model for future studies. It is essential to recognize that AD is a highly complex disease influenced by and affecting diverse systems and processes. Therefore, a comprehensive investigation of the wide range of factors that may contribute to the disease’s initiation and progression is essential. The link between depression and dementia is intricate and multifaceted, as shown in studies indicating that depression may serve as both a risk factor and an early indicator of AD and other forms of dementia, as well as being a frequent issue throughout all stages of dementia. Evidence suggests that experiencing depression earlier in life correlates with an increased likelihood of developing dementia in the future, whereas depression in later life can act as an early sign of the condition [65]. Depression induces structural changes in the brain, including decreased hippocampal volume, impaired neurogenesis, and enhanced neuronal apoptosis, which collectively elevates the susceptibility to AD by exacerbating neurodegenerative processes and accelerating cognitive decline [61,62]. A study involving 1965 participants who had mild cognitive impairment and clinically diagnosed depression revealed that 39.7% progressed to AD over 27 months. The results indicated that individuals with a recent history of depression within the last two years had a significantly higher risk of developing AD compared to those with more distant depressive episodes [66]. Additionally, a recent analysis of 129,410 individuals diagnosed with AD, 390,088 with all-cause dementia, and 3,900,880 age- and gender-matched controls without a history of dementia or depression revealed that the risk of depression was greater than twofold for both men and women with AD [67]. Both AD and depression are related to stress and are significantly influenced by gut microbiota, dietary habits, and physical activity [68]. In addition to endogenous risk factors, AD and depression also have common external risk factors, including exposure to air pollution [69,70]. A study that included 1583 women aged 80 and older without dementia found that living in areas with high levels of fine particulate matter-PM_2.5_ and NO_2_ in late-life might lead to a slight increase in depressive symptoms and could indirectly contribute to a deterioration in episodic memory, highlighting that the adverse effects of air pollution on the connection between depressive symptoms and episodic memory may vary depending on specific pollutants and the age of individuals [71]. Recent research underscored a common single nucleotide polymorphism in the transmembrane protein 106B (*TMEM106B*) gene which was significantly associated with AD and depression, highlighting the need for further exploration of the complex genetic link between AD and depression [72].

The association between AD and diabetes mellitus is also well-established [73]. According to an analysis of 980 patients with mild cognitive impairment categorized by their diabetes mellitus status, diabetes mellitus significantly correlates with cognitive decline and an increased risk of progressing to AD, especially within the first year of cognitive impairment follow-up, as evidenced by accelerating nucleus accumbens atrophy, decreasing gray matter volume, and sulcal depth [63].

Last but not least, shared genetic loci and comorbidities between AD and immune-mediated diseases highlight the significance of the immunoinflammatory hypothesis, suggesting that the immune system may play a common role in both AD and immune-mediated conditions [74]. AD exhibits common pathophysiological mechanisms with some chronic inflammatory conditions, such as rheumatoid arthritis and inflammatory bowel diseases. A key factor in this convergence is the dysregulation of lipid metabolism, which precipitates systemic inflammation and exacerbates tissue degeneration. Furthermore, the activation of the inflammatory cascade plays a pivotal role in the progression of the disease and the emergence of related comorbidities [75]. Researchers underscore the importance of considering the gut microbiota and microbiota–gut–brain axis in the context of the interplay between AD and the immune system [75,76]. Several studies have demonstrated a higher incidence of dementia in inflammatory bowel disease patients. Furthermore, these investigations have also established a correlation between Crohn’s disease, ulcerative colitis, and AD [77,78,79]. A more profound understanding of the microbiota–gut–brain axis and its interplay with AD has the potential to enhance diagnostic and therapeutic approaches. Additionally, such insights may elucidate the mechanisms underlying the condition, particularly concerning inflammation and immune system-related processes.

### 2.5. Infectious Agents

Several infectious agents have been proposed as potential risk factors for AD. Bathini et al. recently reviewed the role of microbial infections as a potential contributor to AD pathobiology, especially concerning sensory dysfunctions, including the olfactory sense or others. In this context, neurotropic pathogens, including bacteria, amoebae, fungi, and viruses, may exploit these sensory nerves as a route of infection and invade the central nervous system [80]. There is growing evidence suggesting that the *Herpes simplex* virus (HSV), *Borrelia burgdorferi*, bacteria that cause Lyme disease, as well as *Porphyromonas gingivalis*, a cause of periodontitis, and *Chlamydia pneumoniae*, bacteria responsible for lung infections, are associated with AD [81,82,83,84].

It is estimated that patients with genital or non-genital HSV infection are approximately 2.56 times more likely to develop any dementia [85]. In comparison to those who do not take medications, patients with HSV infections who used anti-herpetic drugs developed dementia less frequently in the subsequent 10 years. Anti-herpetic medications, including acyclovir, famciclovir, ganciclovir, valacyclovir, and valganciclovir, can reduce the risk of dementia in HSV patients by almost 90.8%. After at least 30 days of treatment, the adjusted hazard ratios were 0.031, 0.042, 0.055, 0.099, and 0.077, respectively. Based on the results of this study, acyclovir was reported as the most effective anti-herpetic drug [85].

While more evidence is required for confirmation, researchers believe that the SARS-CoV-2 virus that causes COVID-19 can invade the brain [86]. Among 36.4% of patients with COVID-19, neurological symptoms were more frequent (45.5%) among those with severe infection. Neurological symptoms of the central nervous system (CNS) include acute cerebrovascular disease, impaired consciousness, ataxia, dizziness, seizures, and headaches. Some patients with COVID-19 displayed neurological symptoms alone rather than the typical symptoms observed in other patients. Therefore, Mao et al. emphasized the neurological symptoms of patients with COVID-19, particularly those with severe illness [87].

Furthermore, several additional infectious agents have been proposed as potential risk factors for AD, although the evidence remains inconclusive and more research is needed. For example, *C. pneumoniae*, a bacterium that has been presumed to traverse the blood–brain barrier and invade the CNS, has been observed in the brains of AD patients [88,89,90]. Additionally, in experimental models of chlamydial infection, reactive astrocytes were observed localizing to Aβ plaques [91]. Chlamydial infection can also increase both β-secretase and γ-secretase activity while decreasing the activity of α-secretase, consequently triggering the amyloidogenic processing of APP [81].

Another infectious agent that can cross the blood–brain barrier and invade the CNS and is associated with AD is *Toxoplasma gondii* [92,93]. In infected wild-type mice, major signs of AD, including Aβ immunoreactivity, pTau expression, neuronal dysfunction, and behavioral alterations, were reported. Toxoplasmosis infection has been demonstrated to induce the accumulation of Aβ plaques and tau hyperphosphorylation in both the hippocampus and the prefrontal cortex [94]. However, several studies observed Toxoplasma strain Type II could reduce Aβ plaque burden by more than 60% and protect against AD [95,96,97]. Jung et al. hypothesized that the protective effect observed might be the result of reduced neurodegeneration and neuroinflammation due to increases in TGF-β and IL-10 [96]. Another hypothesis suggests that the induction of highly phagocytic monocytes due to Toxoplasma infection could reduce Aβ plaque deposition [95]. Briefly, while *T. gondii* is generally considered a risk factor for AD, the relationship is unclear and further research is required to understand the possible mechanisms.

Even though there is limited evidence and the available results are variable, researchers suggest that there might be a link between AD and periodontal disease [98,99]. The underlying biological mechanism might be neuroinflammation due to *P. gingivalis*, bacteria that cause periodontitis [100]. In the study of Wu et al. mice were exposed to 1 mg/kg of lipopolysaccharide from *P. gingivalis* daily for 5 weeks in a row. After 5 weeks, they observed significantly increased microglial interleukin-1β and memory and learning impairments, as well as the accumulation of Aβ in neurons [101]. Moreover, Dominy et al. observed *P. gingivalis* DNA in both the brain and the CSF of individuals diagnosed with probable AD and suggested *P. gingivalis* DNA in the CSF might be a new diagnostic marker for AD. Also, they found that exposure to gingipains, a major virulence factor of *P. gingivalis*, increased degeneration of the neurons compared to the control. After 6 weeks of exposure to *P. gingivalis*, Aβ1–42 levels significantly increased in the mouse brain. Given this, researchers suggested that inhibiting gingipain might be beneficial for AD [82]. These results are significant because patients with AD have worse dental health, meaning they tend to have more caries, mucosal lesions such as candidiasis, and periodontal diseases, as well as poorer the quality and quantity of saliva [102].

Consistent with these studies, in an elderly cohort of 468 participants, the clinical, microbiological, and host response features of periodontitis were associated with MRI markers of atrophy/cerebrovascular disease findings related to AD/AD-related dementia risk, specifically underlining the association of greater levels of periodontitis with lower entorhinal cortex volume and lower cortical thickness in regions implicated in AD [103].

### 2.6. Environmental Factors

The term “exposome” encompasses life-course environmental exposures, including lifestyle factors, from the prenatal period onwards and is highly variable and dynamic; therefore, accurately determining a single individual’s exposure history may be challenging [104]. A systematic review of 4784 studies found moderate evidence linking dementia to environmental risk factors, including air pollution, aluminum, silicon, selenium, pesticides, vitamin D deficiency, and exposure to electric and magnetic fields [105]. An AD exposome has been proposed to address some gaps in understanding environmental contributions to the genetic and nongenetic risk of AD and AD-related dementias [106].

#### 2.6.1. Air Pollution

There is increasing evidence indicating that air pollution is an important factor influencing the aging brain and increased dementia [107]. The causal relationship between late-life exposure to air pollution and dementia risk has been shown with the observation that long-term air quality improvement was associated with lower dementia risk among older women (HRPM_2.5_ 0.80 per 1.78 μg/m^3^, 95% CI 0.71–0.91; HRNO_2_ 0.80 per 3.91 parts per billion, 95% CI 0.71–0.90). The observed effect did not vary by age, education, underlying genetic risks, cardiovascular risk factors, or region [108].

Air pollution encompasses various components, including particulate matter (e.g., PM_2.5_, PM_10_), ozone (O_3_), nitrogen dioxide, sulfur dioxide, carbon monoxide, and black carbon [109]. It would be beneficial to clarify which components are most critical and through which specific metabolic mechanisms they influence this association. A systematic review of longitudinal studies of exposure to airborne pollutants and incident dementia or cognitive decline in adults underlined that greater exposure to PM_2.5_, NO_2_/NO_x_, and CO was associated with an increased risk of dementia [110].

A UK Biobank cohort study enrolling 192,300 participants without dementia investigated the longitudinal associations between air pollution, metabolic signatures, and dementia risk, exploring how air pollution might cause dementia through metabolic pathways. Researchers found 2592 dementia cases linked to air pollution, pinpointing 87 metabolites for PM_2.5_, 65 for PM_10_, 76 for NO_2_, and 71 for NO_x_. With hazard ratios (HRs) for PM_2.5_ of 1.17 (95% CI: 1.12, 1.22), PM_10_ of 1.06 (95% CI: 1.02, 1.11), NO_2_ of 1.16 (95% CI: 1.10, 1.21), and NO_x_ of 1.17 (95% CI: 1.12, 1.22), the metabolic signatures linked to air pollution were associated with an increased risk of dementia. Metabolite-free cholesterol in medium VLDL (M-VLDL-FC) appears to be an important mediating factor [111]. In a cross-sectional study with 176,345 participants aged 60–100 years in northwestern China, long-term exposures to PM_2.5_, PM_10_, and O_3_ were associated with poor cognitive function in the elderly [112].

Particulate matter (PM) is an air pollutant made of liquid and solid particles in the atmosphere that can cause significant damage to human health. Components of PM include polycyclic aromatic hydrocarbons (PAH), nitrates, metals, sulfates, and elemental and organic carbon. According to the WHO, PM_2.5_ (fine PM) is a more significant risk factor compared to PM_10_, especially in long-term exposure [113]. Thus, many studies focused on the effects of PM_2.5_. In a study conducted by Kioumourtzoglou et al. on an elderly population of 9.8 million subjects residing in 50 cities in the northeastern United States between 1999 and 2010, the impact of long-term exposure to PM_2.5_ was investigated. The hazard ratios of AD, dementia, and Parkinson’s disease increased by 1.15, 1.08, and 1.08, respectively, for every 1 g/m^3^ increase in annual exposure to PM_2.5_. Furthermore, a 5 g/m³ increase in annual exposure to PM_2.5_ has been shown to cause an increased risk of AD, dementia, and Parkinson’s disease by factors of 2, 1.46, and 1.44, respectively. The researchers concluded that air pollution is likely to accelerate the progression of neurodegeneration, potentially after the onset of the disease [114]. In an experimental model, Bhatt et al. demonstrated that nine months of exposure to PM_2.5_ at levels below 15 μg/m^3^ led to a significant increase in levels of COX-1 and COX-2 proteins, as well as Aβ and BACE, an enzyme involved in APP proteolysis, in mice brains [115]. Nevertheless, no alterations were observed in microglial activation, hyperphosphorylated tau levels, or tau protein levels. Furthermore, none of these changes were observed in mice exposed to PM for 3 months. Therefore, the researchers emphasized the significance of prolonged exposure [115].

In this context, there are important considerations, such as the variability of the chemical components of PM across different spatial–temporal samplings, as well as the differing chemical combinations of PM. Ard et al. [116] conducted a study evaluating the residential locations of individuals across the years 2002–2012 to estimate their long-term exposure to both PM and probable industrial air pollutants from nearby sources and create individual exposure profiles throughout the decade, focusing on neurotoxic exposures. The researchers analyzed cognitive scores over a decade to understand whether air pollution had any critical impact on cognitive development trajectories. The results demonstrated that participants with higher neurotoxic exposure over time experienced significantly accelerated rates of cognitive decline. Long-term exposure to air pollution is a notable risk factor for AD and Parkinson’s disease. Additionally, *APOE* ε4 allele carriers may experience an elevated risk of developing AD, particularly in environments with high pollution levels [117]. Calderón-Garcidueñas et al. highlighted that exposure to high levels of air pollutants such as ultra-fine PM and PM_2.5_, leads to neuroinflammation and alterations in innate immune responses in critical brain areas in children and young adults [117]. While the *APOE4* effect on CSF Aβ1–42 levels is significant for both genders, its influence on AD risk is more significant in women than in men: Female carriers of the *APOE4* allele exhibit widespread brain hypometabolism and cortical thinning when compared to female non-carriers. In contrast, male *APOE4* carriers demonstrate only a limited cluster of hypometabolism along with regions of cortical thickening relative to their non-carrier counterparts [118]. Air pollution causes neuroinflammation, modifies the innate immune response in the brain, and leads to the accumulation of Aβ42 and α-synuclein beginning in childhood. Another study involving children in Mexico City chronically exposed to high levels of PM_2.5_ and ozone demonstrated that female *APOE4* carriers are more susceptible to air pollution-induced alterations and adverse effects on cognitive function [119]. In this vein, Calderón-Garcidueñas et al. [120] emphasized the critical adverse effects of early life exposures. The researchers indicated that gradual progression of AD starts in childhood and in 99.25% of 134 consecutive autopsies of individuals ≤30 years, the stage of the disease/progression can be identified. It has been shown that 66% of ≤30 years urbanites experience cognitive impairment, and the involvement of the brainstem is reflected by auditory central dysfunction in these individuals. Another noteworthy aspect was that *APOE4* vs *APOE3* carriers had 1.26 times higher odds of committing suicide. PM_2.5_ and nanoparticles produced by combustion and friction significantly contribute to the onset of neuroinflammation and neurodegeneration in young urban populations. Alemany et al. [121] conducted a study with 156 cognitively unimpaired adults at higher risk for AD from the ALFA+ study, examining the relationship between air pollution and AD biomarkers, including CSF levels of Aβ42, Aβ40, p-tau, t-tau, neurofilament light chain (NfL), and cerebral amyloid load. They also evaluated whether *APOE* ε4 influenced these associations. The researchers assessed long-term exposure to NO_2_ and PM to evaluate the impact of residential exposure. The findings indicated that higher exposure to certain pollutants is linked to an increased risk of AD. Specifically, elevated exposure to NO_2_ and PM_2.5_ absorbance was associated with higher levels of brain Aβ deposition as assessed by Aβ PET scans. Additionally, exposure to PM_10_ and PM_2.5_ was positively correlated with higher levels of CSF NfL, which indicates neuronal injury. Notably, higher exposure to NO_2_, PM_10_, and PM_2.5_ is associated with elevated tau-related biomarkers and NfL levels among participants along the Alzheimer’s continuum. Although the presence of the *APOE* ε4 allele did not significantly alter these associations, the effects of air pollution on NfL were more pronounced in *APOE ε4* carriers, and the correlations relating to cerebral amyloid load were stronger in non-carriers.

An increasing amount of research highlights the link between neurodegenerative disorders, such as AD, and exposure to traffic-related air pollution (TRAP). Nevertheless, the specific mechanisms behind this connection are not fully understood. A more detailed examination of the impact of TRAP on hippocampal volume, an important biomarker of neurodegeneration, could help delineate the mechanisms. A study investigating the relationship between TRAP and hippocampal volume in older participants from the UK Biobank found significant interactions between the distance of the participant’s main residence to the nearest major road (DNMR) and the *APOE4* allele that affected hippocampal volume. In particular, a DNMR of less than 50 m, corresponding to chronic high exposure to TRAP, in conjunction with the presence of *APOE4*, was significantly (*p* = 0.01) associated with a decrease of about 2.5% in right hippocampal volume in females aged 60 to 75 years, while the findings for men did not achieve statistical significance. The study’s findings imply that TRAP and *APOE4* may synergistically contribute to neurodegeneration in females. Residing at a greater distance from major roads may help lower the risks of AD and other neurodegenerative diseases for female carriers of *APOE4* [122].

The results of a recent study [123] assessing the association between long-term wildfire and non-wildfire PM_2.5_ exposure and risk of incident dementia showed that in adjusted models, an increase of 1 μg/m^3^ in the 3-year average of wildfire PM_2.5_ exposure was linked to an 18% rise in the likelihood of receiving a dementia diagnosis (odds ratio [OR], 1.18; 95% CI, 1.03–1.34). A similar 1 μg/m^3^ increase in non-wildfire PM_2.5_ exposure was connected to just a 1% rise (OR, 1.01; 95% CI, 1.01–1.02). For wildfire PM_2.5_ exposure, the associations were more pronounced among individuals under 75 years old when they joined the cohort, with stronger associations observed in potentially vulnerable subgroups. In another study, a collection of publicly accessible variables related to environmental pollution, health, social factors, and economic variables were utilized as inputs for a random forest algorithm to create an artificial intelligence model to estimate AD mortality across Italian provinces for five years (2015–2019). The findings indicated that air pollution, particularly O_3_ and NO_2_, was the most significant predictor of AD mortality, underscoring the necessity for further comprehensive research into the relationship between environmental factors and disease [124]. Furthermore, recent studies have shown that air pollution could disrupt gut microbiota [125,126]. When these data are taken together with increasing evidence regarding the role of gut microbiota in regulating bidirectional interactions between the gut and brain through neural, endocrine, and immune pathways [127], more studies are warranted for a clearer understanding of the link between gut microbiota and PM_2.5_.

#### 2.6.2. Toxic Heavy Metals and Other Elements

In a study by Liu et al. [128] the relationship between cognitive impairment and blood levels of toxic elements such as arsenic (As), cadmium (Cd), lead (Pb), strontium (Sr), and vanadium (V) was examined among elderly people living in Chinese communities. Their findings indicated a positive association with cognitive impairment, predominantly associated with Pb; additionally, the presence of the *APOE* ε4 genotype heightened the relationship between cognitive impairment and exposure to Pb and element mixture. Another study [129] sought to identify non-invasive biomarkers for AD by detecting various miRNAs in the buffy coat of blood samples, with four of these also found in the brain tissues of both AD patients and control subjects. Specifically, Pb exposure was negatively associated with hsa-miR-3651, hsa-miR-150-5p, and hsa-miR-664b-3p, while hsa-miR-627 was positively correlated. Furthermore, two of these miRNAs, miR-3651 and miR-664b-3p, exhibited significant differences in expression levels between the brains of AD patients and controls. In research conducted by Wu et al. [130] in *C57BL/6J* and *APP/PS1* models, lead acetate exposure via drinking water from one week prior to mating until offspring reached seven months old resulted in increased levels of p-tau along with reduced mRNA and protein expression of low-density lipoprotein receptor (LRP-1) in both strains. Moreover, early life exposure to Pb was found to accelerate the deposition of Aβ1–42 in the brains of *APP/PS1* mice and lead to abnormal alterations in the proteins Zonula Occludin-1 (ZO-1) and Claudin-5, which are associated with blood–brain barrier junctions.

Aluminum has long been a topic of debate. A cohort study involving 3777 individuals aged 65 or older, conducted in southwest France over 15 years (1988–2003), examined the relationship between exposure to aluminum or silica in drinking water and the risk of cognitive decline, dementia. The findings revealed that cognitive decline was more significant in individuals with a higher daily aluminum intake from drinking water (≥0.1 mg/day) or those living in areas with elevated aluminum exposure. Furthermore, a positive association was noted between high daily aluminum exposure in drinking water and the risk of developing dementia, while a negative correlation was observed with silica intake [131]. In a study by Mirza et al. the brain tissue samples of 12 donors with familial AD were analyzed. The results revealed that 11 out of 12 individuals exhibited at least one tissue sample with a pathologically significant level of aluminum (≥3.00 µg/g dry weight (dw)). A total of nine individuals demonstrated aluminum concentrations exceeding 5 µg/g dw in one or more tissues, while five individuals exhibited aluminum concentrations of at least 10 µg/g dw in one or more tissues [132]. The analysis revealed that approximately 40% of tissues (57/144) exhibited aluminum concentrations that were considered to be pathologically concerning (≥2.00 µg/g dry weight), while approximately 58% of tissues (86/144) demonstrated aluminum concentrations that were deemed to be pathologically significant (≥3.00 µg/g dw). Based on the data corroborated by visual evidence of aluminum within the brain tissue, the researchers suggested that individuals with a genetic predisposition to AD may be more likely to retain and accumulate aluminum within the brain [132]. Another study identified that both familial and sporadic AD patients exhibit considerably elevated aluminum levels within their brains, regardless of age. This finding has prompted researchers to suggest that increased brain aluminum level is not necessarily a consequence of aging [133].

Another element of concern is iron. Becerril-Ortega et al. observed that exposure to 100 mM of iron resulted in 35% elevation in reactive oxygen species (ROS) in glia 24 h after exposure; however, 1- and 10-mM concentrations did not significantly affect the mice. Regarding neurons, exposure to 10 mM iron increased ROS production by 27.2%, while exposure to 100 mM iron caused a rise of 45.7%. This evidence suggests greater sensitivity of neurons to iron than glia. Furthermore, a 24 h exposure to 1 mM iron elevated the Aβ level in neuronal culture, while a 6 h incubation with 1 mM iron increased KPI-APP mRNA expression [134]. Ayton et al. identified a correlation between NFT levels and inferior temporal iron. Their findings indicated that 17% of the impact of NFT on global cognition was driven by these iron levels. However, iron buildup in the brains of patients with advanced pathology acts independently of NFT, significantly affecting global cognition’s decline rate [135]. On the other hand, in a placebo-controlled randomized clinical trial spanning 12 months, the administration of brain-permeable oral iron chelator deferiprone was observed to accelerate cognitive decline in amyloid-confirmed early AD patients, indicating that reduction in iron levels with deferiprone may result in adverse effects in patients with AD [136].

Some other elements, such as copper, have also attracted attention. In their meta-analysis, Li and colleagues observed a notable elevation of copper serum levels in AD patients when compared to a control group of healthy individuals. If this elevation in copper concentration exceeds the capacity of buffers, copper could bind with peptides or proteins and pass through the blood–brain barrier with them, potentially entering the brain [137]. Parthasarathy et al. have demonstrated that copper ions may trigger the production of reactive oxygen species by binding with Aβ fibrils. The complex can cause oxidative damage to neurons through the accumulation of hydrogen peroxide. Researchers have proposed that the presence of copper may be linked with the subsequent pathological events observed in AD [138]. Moreover, Voss et al. observed that an excessive quantity of copper (400 µM) induced tau phosphorylation in human neuroblastoma cell culture in a dose-dependent manner. Furthermore, the administration of zinc acetate, an agent that decreases copper levels, significantly reduced the phosphorylation state of tau in wild-type human tau (hTau) mice compared with untreated hTau mice. While mice treated with zinc acetate performed similarly in a Morris water maze to untreated mice, their performance was superior in a novel object recognition task, indicating improved memory [139]. In view of Cu^2+^ ion chelators as promising agents against AD, Gharai et al. [140] designed and synthesized a dopamine-based molecule that chelates Cu^2+^, inhibits copper-induced amyloid aggregation, and decreases Aβ42-Cu^2+^ complex-mediated cellular toxicity. The ligands have been shown to exhibit the dual properties of dopamine, both as an ROS scavenger and chelator of copper ions.

#### 2.6.3. Pesticides

Epidemiological and laboratory research suggest an association between exposure to neurotoxic pesticides and cognitive dysfunction, including AD [141]. In a study utilizing the Cox proportional hazards model to assess the risk of AD for 26 agricultural activities, higher risks of AD were found in agricultural activities where the use of pesticides were the highest, namely for crop farming (hazard ratio (HR) = 3.72 [3.47–3.98]), viticulture (HR = 1.29 [1.18–1.42]), and fruit arboriculture (HR = 1.36 [1.15–1.62]), while lower risks were found among breeders [142]. Some studies suggest long-term, low-dose pesticide exposure may contribute to AD development. A meta-analysis suggested a positive association between pesticide exposure and AD (OR = 1.34; 95% confidence interval [CI] = 1.08, 1.67; n = 7), confirming the hypothesis that pesticide exposure may be a risk factor for AD [143]. While dichloro-diphenyl-trichloroethane (DDT) was banned in many countries more than 50 years ago because of its long half-life, because of its persistence in the environment, as well as occasional use in some countries, its metabolite p,p’-dichlorodiphenyldichloroethylene (DDE) is still detectable. Richardson et al. reported that patients with AD had 3.8-fold higher serum levels of DDE, a long-lasting metabolite of the organochlorine pesticide DDT, associated with an increased risk of AD [144]. Further mechanistic studies revealed that DDT exposure significantly elevated APP mRNA and protein levels in SH-SY5Y cells, primary neurons, and wild-type C57BL/6J and 3xTG-AD mouse models. Additionally, they identified increased levels of secreted Aβ in SH-SY5Y cells, an effect reversed by the sodium channel antagonist tetrodotoxin. Transgenic flies and 3xTG-AD mice displayed more significant Aβ pathology following exposure to DDT. Moreover, synaptic markers synaptophysin and PSD95 were reduced in the cortices of 3xTG-AD mouse brains [145]. Hayden et al. [146] reported that occupational exposure to pesticides may increase the risk of incident AD, with a slightly higher risk for organophosphate exposure (HR 1.53, 95% CI 1.05–2.23) than organochlorines (HR 1.49, 95% CI 0.99–2.24).

Recently, Bartholomew et al. [147] reported that glyphosate-exposed 3xTg-AD mice exhibited reduced survival rates, along with significant increases in the beta-secretase enzyme (BACE-1) of amyloidogenic processing, Aβ 42 insoluble fractions, Aβ 42 plaque load, plaque size, and p-tau at epitopes Threonine 181, Serine 396, and AT8 (Serine 202, Threonine 205). Pro- and anti-inflammatory cytokines and chemokines were also elevated in the cortical brain tissue of 3xTg-AD and non-transgenic mice and the peripheral blood plasma of 3xTg-AD mice. Moreover, despite the 6-month recovery period, aminomethylphosphonic acid, the major metabolite of glyphosate, was detectable in the brains of exposed groups of both 3xTg-AD and non-transgenic mice in a dose-dependent manner. A recent study conducted in Spain found a positive association between pesticide exposure and the risk of developing AD in individuals residing in areas with high pesticide use, which is evident in the higher odds for AD than in regions with low use (OR: 2.09). In addition, women had the highest odds, with an OR of 2.27, suggesting that women are particularly susceptible to the effects of pesticides [148].

#### 2.6.4. Microplastics and Nanoplastics

Other emerging pollutant groups are microplastics and nanoplastics, which pose potential risks for cognitive impairment and various toxic effects [149]. A significant concern is their ability to cross the blood–brain barrier, potentially contributing to neurotoxicity, dementia, or related effects. Currently, there is limited understanding of the impact on the brain and the pathophysiology/pathways involved in microplastic- and nanoplastic-related adverse health consequences; thus, it is imperative to delve into how these materials can impact brain function, using systematic methods to assess the risks associated with mental health conditions [150]. Research has shown that orally ingested nanoplastics (30–50 nm) can accumulate in the brains of adult mice, leading to cognitive impairment. The preferential uptake of nanoplastics by microglia triggers microglial activation, leading to the dysregulation of hippocampal neuronal activity and the elicitation of inflammatory responses [151]. Results of a recent in vitro study demonstrated that while low-dose PS nanoparticles do not exhibit discernible neurotoxicity, they remarkably expedite the nucleation rate of Aβ40 and Aβ42, prompting heightened Aβ oligomers. Therefore, polystyrene nanoparticles exacerbate the neurotoxicity induced by low-concentration Aβ, leading to evident cell membrane damage and elevated ROS and Ca^2+^ levels [152]. Detailed research is required that broadens the scope of human studies, particularly regarding microplastic and nanoplastic exposure levels, their influence on the gut microbiome and brain health, along with comprehensive clinical evaluations [153].

### 2.7. Tobacco

Current data indicates that smoking may significantly contribute to the development of AD and other neurodegenerative disorders. In a study by Yu et al., exposure to cigarette smoke was associated with decreased anterior corpus callosum thickness, myelin pallor, and white matter degeneration [154]. Moreover, short-term cessation did not reverse these effects. Researchers have proposed that myelin pallor may result from impaired myelin maintenance, damaged myelinated fibers, and demyelination, while white matter demyelination may result from reduced myelin maintenance. After exposure to cigarette smoke, inhibitory effects on the expression of mature and immature oligodendroglial transcription factor (TF) genes, and neuroglial cells were observed, and prolonged exposure increased this inhibitory effect [154].

In a 7-year follow-up study, Choi et al. followed 46,140 individuals, of whom 12,672 were continuous smokers, 20,025 were never smokers, 4175 were short-term abstainers, and 9268 were long-term abstainers. During this follow-up period, 1644 individuals had been diagnosed with AD [155]. In this study, it was observed that long-term quitters and never-smokers had reduced overall risks of dementia as well as reduced risks of developing AD and vascular dementia compared with continuous smokers; consistent with this finding, the overall risk of dementia decreased markedly with decreasing exposure to tobacco. In addition, current smokers have a higher risk of developing dementia. The researchers concluded that smoking should be considered a risk factor for the development of AD, particularly in older people, and that smoking cessation should be encouraged because of the beneficial effects of long-term smoking cessation [155].

Another study found a significant correlation between the frequency of tobacco smoking and relative brain age. Relative brain age is a measure determined by whole-brain anatomical measurements and represents the age of the participant’s brain compared to that of their peers, regardless of biological age [156]. Relative brain age is independent of biological age, and the researchers expected that those with positive relative brain age would have lower cognitive function. People with lower relative brain age performed better on the memory test. It is also interesting to note that the researchers found that those with two *APOE* ε4 alleles had a barely higher relative brain age compared with non-carriers or carriers of only one allele. While the relative brain age of individuals who smoke all or most days is notably higher than that of those who smoke less frequently, there was no significant difference between people who rarely smoke, have only tried smoking once or twice in their lives, or abstain from smoking. In light of these findings, the researchers suggested that the adverse effects of smoking on the brain may be more likely to be seen in people who smoke most days [156].

A study conducted between 1990 and 2021 explored the relationship between tobacco use and the mortality rate of patients diagnosed with AD and related dementias (ADRD). The results demonstrated that tobacco use is positively correlated with an increased death rate in ADRD patients. Researchers highlighted that tobacco use was more strongly associated with mortality rates in ADRD and exerted a more pronounced effect in men compared to women, suggesting that this may be attributable to males being more predisposed to engage in heavier smoking compared to females. Surprisingly, in Cluster A, which primarily included African and Middle Eastern countries, tobacco appeared to offer protective effects. Consequently, researchers have proposed the need for further investigation to gain a deeper understanding of the underlying factors [157].

### 2.8. Alcohol

The effects of alcohol exposure on AD biomarkers and cognitive behaviors have been demonstrated in preclinical experimental models [158,159]. Day et al. found that *APP/PS1* mice exhibited anxiety-related behaviors and increased locomotor activity compared to control mice after 3 weeks of exposure to moderate amounts of ethanol. These changes may lead to impulsive and hyperactive behaviors in ethanol-exposed mice. After 7 weeks of exposure to ethanol, the mice showed some changes in self-care behavior; for example, they had lower nesting values compared to the H₂O controls [158]. The rapid increase in the ethanol concentration in the interstitial fluid following an acute dose of 2.0 g/kg of ethanol suggests that ethanol can easily cross the blood–brain barrier and reach the hippocampal interstitial fluid. While acute ethanol exposure increased Aβ40 levels, there was no change in interstitial fluid Aβ42 levels [158]. Additionally, after ethanol exposure in *APP/PS1* mice, cortical NMDA receptor subunit GluN2B mRNA levels increased. On the other hand, in the hippocampus, ethanol exposure increased the GABAergic subunit GABAA alpha 5 in wild-type mice, while no effect was observed in *APP/PS1* mice. As a result of these changes, the brain’s balance of excitation and inhibition may be disturbed, and ethanol exposure may increase the risk of AD through increased brain excitability. In another study, Hoffman et al. found that after alcohol exposure, the 3xTg-AD mice spent less time in the quadrant of a Morris water maze [159]. Researchers stated that ethanol may impair spatial memory linked to the hippocampus, which is also considered an important issue in AD patients. This study showed freezing behavior to the sign paired with shock in advance, elevated among the alcohol-exposed 3xTg-AD mice compared to the control group exposed to saccharin. Besides behavioral changes, AD biomarkers also changed in particular brain regions. In alcohol-exposed mice, 11 proteins associated with AD, including hexokinase-1 (HK1), syntaxin-binding protein 1 (STXBP), and brain-abundant membrane-attached signal protein 1 (BASP) were found in the amygdala and 12 proteins associated with AD such as ATP synthase F1 subunit delta (ATP5D), enolase 2 (ENO2), and amphiphysin (AMP) were found in the prefrontal cortex. Also, in the hippocampus, tau hyperphosphorylation was significantly increased after 1 month of alcohol exposure [159]. Interestingly, while the former study [158] suggests that acute and single exposure to ethanol selectively increases Aβ40 and does not affect Aβ42, the latter study [159] suggests that after 1 month of alcohol exposure, the Aβ42/Aβ40 ratio increased, suggesting that alcohol exposure selectively elevates Aβ42. A recent study has indicated that increased alcohol consumption could be associated with a reduction in mortality rates from ADRD, establishing that every one-liter increase in pure alcohol consumption among the overall population leads to a decrease in mean ADRD death rates by 0.625 individuals per 100,000 of the population. Furthermore, the study found that the protective effects of alcohol tended to be more pronounced in women than in men. However, researchers cautioned that higher Human Development Index (HDI) levels may diminish this protective effect. Consequently, they advised that countries with high HDIs should consider moderating their alcohol consumption to help lower ADRD mortality rates [157].

### 2.9. Diet

**Figure 4:**
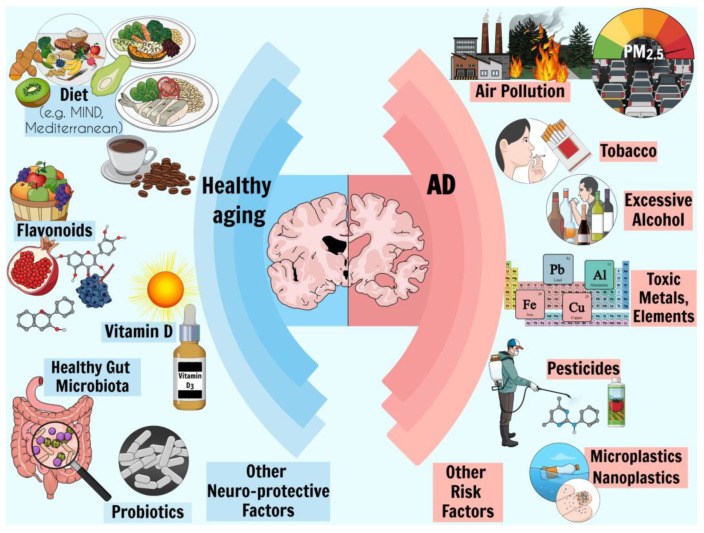
Currently known protective dietary factors such as flavonoids, probiotics, and vitamin D acting on the enhancement of brain resilience and the prevention or inhibition of AD initiation and progression, and the emerging pollutants of the “AD exposome”.

While the prospects in drug research and development for novel, effective, and safer therapeutic options are encouraging, the currently available treatments for AD globally do not offer a cure; rather, these treatments primarily focus on mitigating the symptoms. Consequently, multiple promising areas, including nutrition as a notable modifiable risk factor, have gained significant attention for their potential in the prevention of AD as well as in enhancing quality of life after diagnosis. Nutrition serves as a pivotal element in the promotion of health and the facilitation of effective aging. In accordance with this notion, the concept of “hormetic nutrition” has attracted considerable scholarly interest due to its potential to augment antioxidant and anti-inflammatory pathways. This methodology has a promising effect on biological pathways, particularly the Nrf2 pathway, along with the regulation of detoxification genes and enzymes, including heme oxygenase-1 (HO-1), heat shock protein 70 (Hsp70), sirtuin 1 (Sirt1), glutathione peroxidase (GPx), thioredoxin (Trx), superoxide dismutase (SOD), catalase, and interleukin-10 (IL-10). These factors are vital for providing neuroprotection under various adverse conditions, especially those related to oxidative stress. The concept of “hormesis” is exemplified by several dietary nutrients, such as polyphenols, probiotics, and vitamin D. Hormesis refers to a biphasic dose–response relationship, whereby exposure to substances or stressors that are toxic at high doses may yield beneficial effects at lower doses. This concept has been extensively examined within the domains of toxicology, gerontology, and neuroscience, especially relating to neuroprotection and the therapeutics of disorders associated with the gut–brain axis, underscoring the non-linear relationship between dose and effect, and suggesting that low-level exposure can stimulate adaptive responses that promote health and resilience. Therefore, hormesis can be applied to understanding how certain compounds, when consumed in small amounts, can stimulate adaptive responses that promote health and enhance the resilience of the nervous system. These “hormetic nutrients” at optimal doses play a crucial role in orchestrating cellular stress response pathways, including those related to antioxidant defense, inflammation regulation, and autophagy, which ultimately contribute to neuroprotection against emerging pollutants such as microplastics and nanoplastics, toxic heavy metals, intestinal dysbiosis, free radicals, and infections leading to gut–brain axis disorders [160,161,162,163]. However, as a consequence of their inherent characteristics, hormetic nutrients at high doses can exhibit toxicity and may impair intracellular antioxidant and anti-inflammatory pathways, potentially contributing to the onset and progression of various cognitive disorders. Therefore, considering the critical role of dose as a determining factor for efficacy and toxicity, the hormetic and neuro-adaptive responses elicited by these nutrients in enhancing endogenous redox defense signaling are emerging as a promising approach for both preventive and therapeutic interventions in neurodegenerative disorders linked to oxidative damage and toxicity. It is essential to conduct studies aimed at identifying the optimal dosage required to confer neuroprotective effects in the context of neurodegeneration, which is vital for ensuring brain health and mitigating neurotoxicity [162,163,164]. Figure 4 depicts the currently known protective factors, such as flavonoids, probiotics, and vitamin D, proposed to act as neuroprotectors that, in low doses, upregulate endogenous antioxidant pathways to enhance brain resilience and prevent or inhibit AD initiation and progression, and the emerging pollutants of the AD exposome.

In this context, diverse dietary interventions are being evaluated. A recent exploratory review has highlighted the significant role of diet in AD, particularly emphasizing the potential benefits of various dietary patterns, including the Mediterranean, Dietary Approaches to Stop Hypertension (DASH), and the Mediterranean–DASH Intervention for Neurodegenerative Delay (MIND) diets [165]. A meta-analysis indicated that adherence to the Mediterranean diet, recognized for many benefits over centuries, correlates with a 13% reduction in the incidence of AD and Parkinson’s disease [166]. Cherian et al. conducted an analysis of patients who suffered strokes and subsequently followed a specific dietary regimen, namely the MIND diet, over an average duration of 5.9 years [167]. The MIND diet incorporates a variety of food groups, such as fish, whole grains, vegetables, wine, nuts, olive oil, and poultry, paralleling many components of the Mediterranean diet, with specific refinements according to both epidemiological and animal studies aimed at enhancing neuroprotective effects. The study demonstrated that adherence to the MIND diet significantly attenuated the deterioration of semantic memory and overall cognitive functioning, especially in a subset of participants identified as *APOE* ε4 carriers, comprising 16% of the total sample. Results showed that populations with higher *APOE* ε4 carrier prevalence adhered more to the MIND diet than those with lower prevalence. Furthermore, individuals possessing a higher level of educational attainment, in conjunction with a greater frequency of engagement in cognitive and physical activities, exhibited enhanced adherence to the MIND diet when compared to their peers. The findings of the study indicated no correlation between a slower cognitive decline and either the Mediterranean diet or the DASH diet. The authors postulated that the MIND diet may prove to be more efficacious than the Mediterranean diet in mitigating cognitive decline post-stroke, as it is explicitly formulated for the promotion of brain health [167]. Hosking et al. conducted a 12-year follow-up study revealing that higher adherence to the MIND diet correlated with a 19% reduction in the risk of developing dementia or mild cognitive impairment. Furthermore, individuals in the highest tertile exhibited a 53% lower risk of cognitive impairment compared to those in the medium or low consumption tertile. In contrast, no significant association was observed between cognitive decline and the Mediterranean diet (including the Greek Mediterranean diet and the 9-point Mediterranean diet) following covariate adjustments. Consequently, these findings suggest that the MIND diet is more effective in mitigating the risk of dementia [168]. A cross-sectional study showed that higher scores on the Chinese-adapted MIND diet were positively correlated with improved cognitive function and a lower risk of developing mild cognitive impairment among middle-aged and older Chinese adults. Specifically, for each unit increase in the diet score, the prevalence decreased by 11%, highlighting the potential benefits of the MIND diet for cognitive health [169]. In contrast, a two-site, randomized, controlled trial involving 604 participants who exhibited no cognitive impairment but possessed a familial predisposition to dementia revealed that MRI alterations in white matter hyperintensities, hippocampal volumes, and total gray and white matter volumes were analogous between the participants adhering to the MIND diet and those on a control diet that incorporated a modest caloric restriction over a three-year period [170]. An investigation by van Lent et al. explored the association between Dietary Inflammatory Index (DII) scores and the incidence of all-cause dementia as well as AD-related dementia within a population of 1487 individuals from the Framingham Heart Study Offspring cohort, monitored over an extensive follow-up duration of up to 22.3 years. The authors found a significant correlation between elevated pro-inflammatory DII scores and heightened rates of both all-cause dementia and AD dementia. Furthermore, they posited that dietary patterns characterized by lower DII scores could play a pivotal role in the potential prevention of dementia in later life [171]. An experimental mouse model study conducted by Graham et al. mimicking the eating patterns typical of Western populations, characterized by high levels of sugar, carbohydrates, and fats, alongside a lower intake of essential vitamins, compared to a control group given a diet rich in protein and low in total fat, saturated fatty acids, and cholesterol, revealed that after an 8-month period, there was a significant reduction in the number of hippocampal neurons in mice fed the Western-style diet compared to those in a control group. Additionally, levels of Aβ42 and amyloid plaque counts were increased. The researchers indicated that the Western diet might trigger neuroinflammation, as there was a significant increase in allograft inflammatory factor 1 (IBA1) and glial fibrillary protein (GFAP) in the entorhinal cortex and hippocampus, areas severely impacted by AD [172]. In another study examining the ketogenic diet model, *APP/PS1* mice exhibited notable enhancements in cognitive function alongside a significant reduction in amyloid plaque formation and pro-inflammatory cytokine levels. Furthermore, this diet was found to promote the signaling pathway of nuclear factor-erythroid 2-p45 derived factor 2/heme oxygenase-1 while simultaneously inhibiting the nuclear factor kappa B pathway [173].

Higher flavonol consumption through food has been suggested to be associated with a lower risk of AD [174]. Participants with the highest flavonol consumption had a 48% lower risk of AD than those with the lowest. Also, the members of the flavonol class, such as kaempferol, myricetin, and isorhamnetin, were evaluated, and these are correlated with lowering AD risk by 50%, 38%, and 38%, respectively, for participants with the highest flavonol consumption. Kaempferol can be found in tea, broccoli, and spinach; myricetin in tomatoes, tea, and wine; and isorhamnetin in olive oil, tomato sauce, and pears [174].

Another point of interest that merits careful examination is the impact of caffeine consumption on both humans and experimental models. Current data shows that consuming moderate amounts of caffeine through coffee and tea regularly offers beneficial effects and alleviates the risk of AD. A recent meta-analysis concluded that the relationship between the risk of dementia/AD and tea consumption was linear; a significantly decreased risk of dementia for each cup per day increase in tea consumption was noted, while a non-linear protective relationship was found between coffee intake (one to three cups per day) and dementia [175]. In a study conducted to assess the effects of coffee and tea consumption on the risk of dementia development among individuals with hypertension, Cox-proportional risk modeling in 453,913 participants from a UK biobank revealed a J- and U-shaped association between all-cause dementia risk and the consumption of coffee and tea, respectively. The results showed that hypertensive patients drinking 0.5–1 cup of coffee or 4–5 cups of tea per day experienced the lowest risk of dementia [176].

The potential of herbs and their ingredients/constituents have been extensively examined [177,178], including well-known species such as *Ginkgo biloba* [179] or *Curcuma longa* [180]. Among the proposed mechanisms of protection against Aβ-induced cognitive decline include inhibitory or mitigating effects on Aβ accumulation, oxidative stress, tau hyperphosphorylation, inflammation, synaptic damage, and neuronal apoptosis in the cortex and hippocampus during early and late AD [177]. A recent study that examined safety, stability, and transport across the blood–brain barrier and the pharmacological effects of a nanoformulation of curcumin encapsulated within an H-Ferritin nanocage revealed enhanced inflammatory responses in peripheral blood mononuclear cells of AD patients and mild cognitive benefits in a 5xFAD mouse model. Additionally, the curcumin-containing nanoformulation demonstrated a reduction in both microgliosis and astrogliosis [180].

### 2.10. Vitamin D Deficiency

In their meta-analysis, Shen and Ji [181] reported that participants with vitamin D deficiency (25-hydroxyvitamin D level < 50 nmol/L) had a 21% higher risk of developing AD compared to participants with normal levels of vitamin D (25(OH)D level > 50 nmol/L). The researchers stated that while more evidence is needed, current data show that vitamin D deficiency is associated with AD and dementia [181]. In a prospective cohort study, lower serum vitamin D levels were shown to exacerbate Aβ-associated neurodegeneration, whereas higher levels may help reduce it in nondemented older adults [182]. In a 12-year follow-up study, Feart et al. found that participants with 25-hydroxyvitamin D deficiency had almost three times more risk of developing AD and faster cognitive impairment. Also, researchers stated that there is a clear and significant link between vitamin D deficiency and AD, even more than for *APOE* ε4; thus, maintaining 25-hydroxyvitamin D levels at 50 nmol/L or higher may be a viable approach to decrease the risk of AD and protect brain health [183]. These studies have led researchers to evaluate the possible role of vitamin D supplements in improving memory and cognitive functions. Morello et al. have observed that vitamin D supplementation in the early stages of AD may help improve working memory in mice without decreasing astrogliosis, which is linked with Aβ production or amyloid concentration [184]. Consistently, in the early stages of AD, vitamin D deficiency resulted in a remarkable elevation of amyloid plaques both in the cortex and the hippocampus, although, in later stages, this effect could not be seen. Furthermore, vitamin D supplements improved hippocampal neurogenesis, which is diminished in the early stages of AD, by considerably increasing cell proliferation in the dentate gyrus, resulting in an increase in the generation and differentiation of neural progenitor cells. In the later stages of AD, vitamin D deficiency severely reduced the growth and differentiation of neurons and cell proliferation, yet high levels of vitamin D supplementation could not improve neurogenesis and progenitor or stem cell proliferation in these stages [184]. Also, a recent study suggested that vitamin D significantly inhibited both tau protein phosphorylation and Aβ aggregation, while ameliorating cognitive performance in AD rats [185]. A study with 12,388 participants showed three forms of vitamin D (ergocalciferol, cholecalciferol, and calcium–vitamin D) intake were related to a 40% reduction in the prevalence of dementia. All three forms of vitamin D were effective in decreasing dementia risk with different percentages; meanwhile, no significant differences in HR across formulations were observed [186], which was inconsistent with previous data stating that vitamin D3 is more efficacious at raising serum 25(OH)D concentrations than is vitamin D_2_ [187]. Interestingly, while vitamin D intake lowered the dementia risk of both *APOE* ε4 carriers and non-carriers, a more prominent effect was shown with non-carriers [186].

On the contrary, the results of an animal model and human cohort study caution against prolonged use of vitamin D by AD patients [188]. In the population-based longitudinal study part, vitamin D_3_ supplementation for over 146 days/year was reported to lead to 2.17 times the risk of mortality among participants with pre-existing dementia. Dementia-free participants taking vitamin D3 supplements over the same duration were 1.8 times more likely to develop dementia than those not taking the supplements. In the *APP/PS1* mice model, vitamin D supplementation increased Aβ deposition, exacerbated AD, and enhanced non-genomic vitamin D receptor/p53 complex in AD brains. Thus, it was suggested that vitamin D deficiency may be an early feature or an outcome of AD rather than a cause [188]. In addition, as vitamin D receptor gene polymorphisms could affect the response to vitamin D supplementation, the supplement’s effectiveness may differ across individuals, potentially limiting its applicability [189].

### 2.11. Gut Microbiota

The role of the “microbiota–gut–brain axis” in AD has been extensively reviewed in recent publications [190,191,192]. Moreover, fecal microbiota transplantation from a healthy donor to an AD patient has been shown to provide promising results, including improved cognition and memory [193]. The efficacy and safety of microbiota-based interventions for AD necessitate further clinical trials as well as other points to consider (e.g., potential interactions with drugs) and long-term studies involving more extensive and diverse populations. Current methods for modulating gut microbiota include probiotics, prebiotics, fecal microbiota transplantation, and antibiotics. Although preliminary research indicates the potential benefits of gut microbiota modulation for AD, translating these microbiome-based therapies into clinical practice is complicated due to the intricate nature of the microbiota–gut–brain axis and the possibility of both positive and adverse effects from interventions. Advanced research should focus on clarifying the causal relationships between gut microbiota and AD, investigating the molecular mechanisms behind neuroinflammation regulation in the microbiota–gut–brain axis, identifying additional gut microbiota-related biomarkers for AD, and working on the discovery of more effective, personalized therapies based on gut microbiota modulation. The adaptability of the human gut microbiome presents a promising avenue for precise/personalized microbiota-based treatments for AD [194].

A study conducted by Zhuang et al. [195] identified significant alterations in the gut microbiota of AD patients compared to healthy controls, suggesting a possible association with AD pathogenesis. Specifically, a decline in *Bacteroidia*, *Negativicutes*, *Lanchnospiraceae*, and *Veillonellaceae* was observed, while *Lactobacillaceae*, *Bacilli*, *Enterococcaceae*, and *Actinobacteria* increased in the AD group as compared with the controls. Although no notable difference in *Firmicutes* levels was found, the ratio of *Firmicutes* to Bacteroidetes was altered between the two groups, indicating a significant imbalance in the gut microbiota of AD patients. The authors emphasized that brain health is influenced by overall bodily health, proposing that AD may not solely be a brain disorder. They further suggested that modulating gut microbiota through personalized dietary interventions could help prevent AD. A cohort study of Chinese participants identified significant differences in the microbiome between AD patients, those with pre-onset amnestic mild cognitive impairment, and healthy controls (HC). The study showed that the individuals diagnosed with AD exhibited a marked reduction in fecal microbial diversity, suggesting significant alterations in their gut microbiome composition. Notably, there was a strong correlation between the clinical severity scores of these AD patients and the abundance of altered microbiomes. In examining specific bacterial taxa, researchers found a significant decline in the proportion of the phylum *Firmicutes*. In contrast, there was a substantial increase in the levels of *Proteobacteria* among the AD patients compared to healthy controls. Particular attention was drawn to the increasing prevalence of *Gammaproteobacteria*, *Enterobacteriales*, and *Enterobacteriaceae*, which were found to be progressively increased from healthy individuals to those with amnestic mild cognitive impairment and to AD patients. The Kyoto Encyclopedia of Genes and Genomes (KEGG) results showed that the increased modules were related to glycan biosynthesis and metabolism in AD and amnestic mild cognitive impairment patients, whereas the decreased pathways were associated with the immune system in AD patients [196]. Yıldırım et al. conducted a cohort study utilizing machine learning techniques to analyze the gut microbiota composition in stool samples from 47 patients with AD, 27 patients with mild cognitive impairment, and 51 non-demented control subjects [197]. Their findings revealed a stratified community structure in the gut microbiota along the AD continuum, primarily characterized by *Prevotella* and *Bacteroides*, alongside various subnetworks of other taxa. Notably, the study revealed a negative correlation between AD and the presence of *Roseburia*, *Lactobacillus*, and *Fusicatenibacter*. Haran et al. [198] enrolled 108 elderly people in a prospective cohort study and followed them for up to 5 months, collecting longitudinal stool samples for metagenomic sequencing and in vitro T84 intestinal epithelial cell functional assays for P-glycoprotein (P-gp) expression. The microbiome pattern of AD patients was characterized by a lower relative abundance of butyrate-producing species and higher levels of taxa causing proinflammatory states compared to those with no dementia. This pattern may adversely affect intestinal epithelial homeostasis, leading to lower expression of P-gp. Integrated functional studies with machine learning approaches to identify bacterial species differentiating the microbiome of AD elderly people from those without dementia appear to provide promising predictors of the dysregulation of the P-gp pathway. Angelucci et al. have suggested that any factor causing harm to gut microbiota balance, such as antibiotics, diet, or infections, can trigger AD and have emphasized that groups such as the elderly are more at risk as their immune systems weaken. Antiviral agents against HSV1 and treatment against the *H. pylori* infection are thought to have beneficial effects on cognitive functions, while the impact of antibiotics depends on their type and the microbiome’s role in AD pathogenesis [199]. Another study in rats [200] investigated the effect of antibiotics and probiotics on gut microbiota, evaluating the effects of ampicillin, an antibiotic that can trigger colon inflammation, and *Lactobacillus fermentum* strain NS9. Ampicillin significantly decreased *Lactobacillus* and *Bacteroides* while increasing *Firmicutes* and *C. coccoides* in the gut microbiota of rats. After NS9 administration, *Lactobacillus* levels increased while *C. coccoides* and *Firmicutes* levels decreased. After ampicillin administration, anxiety-like behavior and impairments in learning behaviors were observed, and NS9 application could not reduce this impairment. In a Morris water maze, control rats that consumed drinking water without ampicillin or *L. fermentum* NS9 spent 35%, and NS9-administered rats spent 30% of their time in the quadrant, while rats treated with ampicillin spent 25% of their time in the quadrant, implying that NS9 administration improved memory impairment caused by ampicillin. Additionally, ampicillin decreased levels of NMDA receptors, while NS9 inhibited the decrease and successfully kept NMDA receptor levels in a normal range [200].

Antibiotic usage affects AD pathology through the gut microbiota by changing its composition [199]. As AD is linked with neuroinflammation, antibiotics can be beneficial via their anti-inflammatory effect [201]. Yet, using antibiotics with a broad spectrum for an extended period can be detrimental by causing intestinal dysbiosis. Researchers hypothesize that different results from studies might be because of the durations of the studies and differences in antibiotic preferences [202]. For instance, exposure to an antibiotic mixture including streptomycin, clindamycin, and ampicillin for 3 weeks resulted in impairment in spatial memory, while there have also been studies showing that antibiotics improve memory via different mechanisms, such as reducing tau hyperphosphorylation, plaques, Aβ deposits, or inflammatory markers [201,203,204,205,206]. Other results from these studies with the antibiotics and species discussed are summarized in Table 3.

In a similar but broader approach, the synbiotics defined as a “mixture comprising live microorganisms and substrate(s) selectively utilized by host microorganisms that confer a health benefit on the host” [207] are also of interest. In a transgenic humanized Drosophila melanogaster model of AD [208], a novel synbiotic containing three probiotic strains and a polyphenol-rich prebiotic effectively increased survivability and motility, reduced Aβ-deposition, and restored acetylcholinesterase activity.

## 3. Treatment and Management

Today, no clinical trial data confirm that any interventions can prevent or treat dementia efficiently. For the behavioral and psychological symptoms of dementia, individualized management strategies are used. In view of the pathological mechanisms, currently available treatment strategies include cholinesterase inhibitors, memantine, and anti-amyloid immunomodulators (Table 4). Anti-Aβ application appears as a requirement; however, current data show that it may not be sufficient to have a robust clinical benefit as a monotherapy [209]. Moreover, novel treatment approaches for AD, including CRISPR/Cas9 system [210] and nanoparticle/nanocarrier-mediated drug delivery systems [211] have been investigated and detailed reviews are available. An approach to convert *APOE4* to *APOE3* by gene editing, using a small-molecule structure corrector, ameliorated the detrimental effects, therefore correcting the pathogenic conformation of *APOE4* may serve as a viable therapeutic approach for *APOE4*-related AD [212]. Recent research has highlighted the emergence of other promising fields. A novel *RELN-COLBOS* variant (H3447R) has been linked to resilience against presenilin-linked autosomal-dominant AD and has been validated in a transgenic mouse model. Reelin, an extracellular glycoprotein produced by GABAergic neurons in the hippocampus and cortex, is essential for neurodevelopment, neurogenesis, and neuronal plasticity. Key components of the Reelin pathway are associated with *APOE4*, Aβ, and tau protein pathways, suggesting that they could serve as biomarkers and potential therapeutic targets for AD [213]. Another important aspect is that since lipids are the primary constituents of the brain, any disruption in them could significantly contribute to AD. Clinical studies focused on lipidomics and metabolomics have indicated changes in various lipid classes in the brain in the early stages of AD. Over decades of research, intricate relationships between lipid metabolism and core pathogenic mechanisms of AD—such as amyloid formation, energy deficits, oxidative stress, neuroinflammation, and myelin degeneration—have been uncovered. Numerous clinical studies have clearly illustrated the alterations in lipid species and lipid metabolism involved in the pathogenesis and progression of AD. Significantly, many lipid-related alterations appear during the early phases of the disease, and very recently, high LDL cholesterol has been included in the list of midlife modifiable factors [18]. This implies that imbalances in lipid dyshomeostasis could serve as triggering factors for the disease, likely connected to processes like amyloidogenesis, synaptogenesis, and hypometabolism, thus underscoring the importance of early interventions focused on lipid-centered strategies to enhance treatment effectiveness [214]. In a study aimed at conducting a lipidomic analysis in specific regions of the brain (cerebellum, amygdala, hippocampus, and entire cortex) from wild-type (WT, n = 10) and APPswe/PS1dE9 transgenic (TG, n = 10) female mice aged 5 months, representing a model of early AD, alterations in lipid composition were investigated; notable differences in some lipids were found to be statistically significant between the experimental groups in the cerebellum (n = 68), amygdala (n = 49), hippocampus (n = 48), and cortex (n = 22). Moreover, specific lipids (n = 15) from the glycerolipid, phospholipid, and sphingolipid groups showed statistically significant differences across multiple brain regions between WT and TG. A selection of lipid variables was conducted to create a multivariate approach to evaluate their discriminative capabilities, revealing high diagnostic indices, particularly in the cerebellum and amygdala (sensitivity 70–100% and specificity 80–100%) [215]. Furthermore, Grayson et al. [216] assessed peripheral immune system alteration in an aging cohort at presymptomatic and early symptomatic developmental stages of AD. The researchers proposed that the cognitive status of amyloid-positive participants may be assessed by quantitating the number of exhausted CD4+ and CD8+ T cells. Those with mild cognitive impairment also exhibited an increase in differentiated T cells, as well as myeloid and plasmacytoid dendritic cells in their blood. Given the link between inflammation, infection, and cognitive decline, rejuvenating these cells has been suggested as a potential treatment for AD.

Artificial intelligence to assess and provide a broader evaluation of all proposed mechanisms of AD pathophysiology, along with current treatments and patient profiles as defined by some recent publications [217,218,219], may provide an integrative approach to this emerging global problem. In this context, as already performed in other diseases such as cancer or HIV treatments, multi-target/multi-drug combination therapy to overcome the complex pathology of AD utilization may be of use [209]. Overall, in view of the current trend regarding statistics, a comprehensive approach is warranted to focus on the prevention and treatment of AD to improve dementia care. Identification/characterization of potential risk factors that may have benefits in prevention or risk reduction, as well as the clarification of keystones of disease mechanisms, are essential.

**Table 4:** Currently known/available medications in AD [220,221].

**Pharmacologic Category: Cholinesterase Inhibitors**			
**Donepezil** 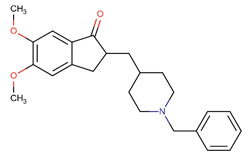 Approval History: 1996: Initial U.S. approval 2014: extended release, combined with memantine 2022: transdermal delivery system	**Rivastigmine** 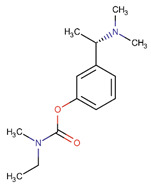 Approval: 2000	**Galantamine** 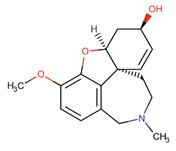 Approval: 2001	**Benzgalantamine** 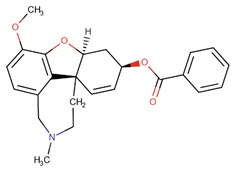 Approval: 2024
**Labeled Indication:** Treatment of mild, moderate, or severe dementia of the Alzheimer’s type.	**Labeled Indication:** **Oral:** Treatment of mild to moderate dementia of AD type. **Transdermal:** Treatment of mild, moderate, and severe dementia of AD type.	**Labeled Indication:** Treatment of mild to moderate dementia of AD type.	**Labeled Indication:** Treatment of mild to moderate dementia of AD type.
▪ A piperidine derivative acetylcholinesterase (AChE) inhibitor for managing the symptoms of AD and other types of dementia. ▪ No known effect in alteration of progression of AD. ▪ Mechanism of action: Selectively and reversibly inhibits the AChE, enhances cholinergic transmission, thus relieves the symptoms. Other possible mechanisms of action include opposition of glutamate-induced excitatory transmission via downregulation of NMDA receptors and regulation of amyloid proteins, along with the inhibition of various inflammatory signaling pathways, exerting neuroprotective effects. ▪ Should be used with caution in patients with risk factors for rhabdomyolysis, peptic ulcer, respiratory disease, urinary tract obstruction, or a history of seizures.	▪ A parasympathomimetic or cholinergic agent for the treatment of mild to moderate dementia of the Alzheimer’s type. ▪ Mechanism of action: Selectively and reversibly inhibits both butyrylcholinesterase and AChE in the brain, preventing the hydrolysis of acetylcholine, thus leading to an increased concentration of acetylcholine at cholinergic synapses. ▪ May cause CNS depression, which may impair physical or mental abilities. It may exacerbate or induce extrapyramidal symptoms. Use with caution in patients with risk factors for rhabdomyolysis, peptic ulcer, respiratory disease, urinary tract obstruction, or a history of seizures.	▪ A tertiary alkaloid extracted from botanical sources such as *Galanthus nivalis.* ▪ No known effect in alteration of the progression of AD. ▪ Mechanism of action: Reversible, competitive inhibitor of AChE. Blocks the breakdown of acetylcholine in the synaptic cleft, enhancing cholinergic neuron function and signaling—also, an allosteric modulator of nicotinic receptor (as a dual mechanism of action). ▪ May cause CNS depression, extrapyramidal effects, vagotonic effects, weight loss, and skin reactions. Use with caution in patients with cardiac conduction abnormalities, hepatic/ renal impairment (not recommended in severe impairment), peptic ulcer disease: respiratory disease, urinary tract obstruction, a history of seizure disorder, particularly in elderly patients with low body weight and/or serious comorbidities.	▪ A prodrug of galantamine. ▪ Gastrointestinal adverse effects are the most frequently reported side effects in patients undergoing treatment with cholinesterase inhibitors and are often a reason for treatment discontinuation. As a prodrug, benzgalantamine remains inert as it passes through the stomach, thereby avoiding many of the gastrointestinal effects associated with peripheral cholinesterase inhibition. ▪ May cause CNS depression, extrapyramidal effects, vagotonic effects, weight loss, and skin reactions. Use with caution in patients with cardiac conduction abnormalities, hepatic/renal impairment (not recommended in severe impairment), peptic ulcer disease: respiratory disease, urinary tract obstruction, and a history of seizure disorder.
**Pharmacologic Category:** **NMDA receptor antagonist**	**Pharmacologic Category: Monoclonal Antibodies**		
**Memantine** 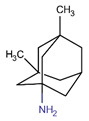 Approval: 2013	**Aducanumab (BIIB037)** Approval: 2021 (accelerated FDA approval)	**Donanemab** Approval: 2024	**Lecanemab** Approval: 2023
**Labeled Indication:** Treatment of moderate to severe dementia of the Alzheimer’s type.	**Labeled Indication:** Treatment of AD in patients with mild cognitive impairment or mild dementia stage of disease	**Labeled Indication:** Treatment of AD; initiated in patients with mild cognitive impairment or mild dementia stage.	**Labeled Indication:** Treatment of AD; to be initiated in patients with mild cognitive impairment or mild dementia stage of disease.
▪ NMDA receptor antagonist used in the management of AD. ▪ Blocks the effects of glutamate, a neurotransmitter in the brain that leads to neuronal excitability and excessive stimulation in AD. ▪ Rare severe skin reactions, such as Stevens–Johnson syndrome, have been observed; patients should promptly report any skin issues. Exercise caution in individuals with heart disease, serious liver and/or kidney impairments, or seizure disorders. A clinical trial has noted a deterioration in corneal conditions. The elimination of the drug is significantly decreased in alkaline urine; therefore, caution is advised when using alkalinizing medications, making dietary modifications, or managing patient conditions that might raise urine pH.	▪ A recombinant monoclonal immunoglobulin gamma 1 (IgG1) antibody targeting extracellular Aβ plaques in the brain. Known as the first “disease-modifying drug” for AD. ▪ First derived from patients with slow or absent cognitive decline, and phase 1b clinical trial data have shown patients treated with aducanumab show a reduction in Aβ plaques. Based on Mini-Mental State Examination and Clinical Dementia Rating (CDR), patients taking aducanumab showed signs of slowing progression; however, these data were controversial. ▪ In January 2024, Biogen announced that it would discontinue the development and commercialization of aducanumab to prioritize the marketing of lecanemab. ▪ May lead to amyloid-related imaging abnormalities (ARIA), defined as “ARIA with edema” (ARIA-E) and “ARIA with hemosiderin deposition” (ARIA-H). Patients who are homozygous for the *APOE* ε4 genotype (~15% of AD patients) receiving treatment with this class of drugs, such as aducanumab, exhibit a greater incidence of ARIA, including symptomatic, serious, and severe radiographic ARIA, compared to those who are heterozygotes or noncarriers.	▪ A humanized IgG1 monoclonal antibody directed against insoluble N-truncated pyroglutamate Aβ, which is found in the brain amyloid plaques that contribute to the pathophysiology of AD. ▪ It is targeted against the insoluble, modified, N-terminal truncated form of the β-amyloid present only in brain amyloid plaques called pyroglutamate Aβ. Upon binding to pyroglutamate Aβ at position 3 (pGlu3-Aβ, AβpE3), donanemab promotes plaque removal through microglial-mediated phagocytosis. ▪ Donanemab may trigger infusion reactions, during infusion or within 30 min following infusion. These include chills, erythema, nausea, vomiting, difficulty breathing, dyspnea, sweating, BP changes, headache, and chest pain. The highest incidence of infusion reactions is observed during the initial four infusions.	▪ A recombinant humanized IgG1 monoclonal antibody directed against aggregated soluble and insoluble forms of Aβ. Aβ peptides exist in various conformational states, including soluble monomers, soluble aggregates of increasing size, and insoluble fibrils and plaque. Soluble Aβ aggregates such as Aβ protofibrils are more neurotoxic than monomers or insoluble fibrils. ▪ Lecanemab preferentially targets soluble aggregated Aβ and works on Aβ oligomers, protofibrils, and insoluble fibrils. In clinical trials, it significantly reduced brain Aβ plaques compared to placebo. ▪ Lecanemab may trigger infusion reactions; symptoms include fever, flu-like symptoms (chills, generalized aches, shakiness, joint pain), nausea, vomiting, hypotension, hypertension, and oxygen desaturation. The greatest likelihood of experiencing infusion reactions is during the initial infusion.

## 4. Conclusions

Today, with only a limited number of drugs available, AD remains one of the deadliest diseases. The lack of a radical treatment option for this disease renders prophylactic measures more critical. To develop more effective approaches, it is crucial to understand various environmental, genetic, and epigenetic risk factors associated with AD. In this study, we undertook a comprehensive review of the existing literature on the issue, focusing on a wide range of risk factors, including tobacco, alcohol, PM, infectious agents, diet, and several diseases. AD, diabetes mellitus, dementia, and other diseases may influence each other, share common risk factors, and act as risk factors for one another, making the connection between AD and these diseases intricate and reciprocal. Additional research is vital to enhance our comprehension of how these conditions relate to each other, which could ultimately benefit individuals dealing with one or both disorders, especially in view of critical implications for prevention and treatment. A deeper insight into common risk factors may help develop targeted preventive measures while uncovering shared underlying mechanisms, which could provide new opportunities for drug repurposing studies. Given the significant economic burden that AD places on families and the global drug development landscape, a thorough understanding of the relationship between AD and intrinsic/extrinsic factors might be of use in developing more precise prevention and treatment strategies, thereby improving cost-effectiveness in patient care and lessening the financial burden of therapeutic development.

Long-term neurologic outcomes should not be disregarded compared to other infectious agents, such as coronavirus, which has only recently emerged. Several studies have indicated that the risk of developing AD is increased among older people in the context of COVID-19, which suggests that the prevalence of AD may be affected globally as a consequence of the pandemic [222]. Further research is required to ascertain the long-term effects of this condition on cognitive functions, particularly in severe cases. Another issue that would benefit from further investigation is the potential association between periodontal diseases and AD. Individuals enrolled in studies should undergo a comprehensive assessment to determine whether they have undiagnosed cognitive impairments [99]. This review also briefly discusses the potential benefits and risks associated with using antibiotics in the context of AD, as well as the mechanisms of these effects. Current data highlight the importance of considering the type and duration of antibiotic use. Furthermore, the significance of nutrition is emphasized. Current pertinent studies revealed that the MIND diet may offer the optimal dietary pattern for AD patients, while the Western diet has been shown to exert adverse effects.

The current study acknowledges specific limitations. It is crucial to recognize that AD research is inherently dynamic; therefore, as new evidence emerges, the conclusions drawn in this review may become outdated, particularly in fields such as genetic research, focus on diet, air quality improvements, and other risk factor interventions, as noted during the preparation of this study. Over time, some risk factors may gain prominence, while others may diminish in significance. In addition, this review employs a narrative style, contrasting with the more structured approach of a systematic review. We examined studies that employ various methodologies, including case-control studies, cohort studies, and meta-analyses. This methodological diversity may complicate the comparison of results across studies, and potential inconsistencies in study designs and outcomes could be misleading in certain contexts. To address this issue, we conducted the literature search continuously, with the collaboration of both authors. Since the review’s scope was constrained by the availability of studies published in English, this limitation may inadvertently exclude relevant publications in other languages. Therefore, it is essential to interpret and compare both historical and contemporary studies with caution.

Overall, to reduce the prevalence and mortality associated with AD, it is essential to identify the risk factors and implement effective strategies to mitigate their impact. A deeper insight into prophylactic methodologies and prospective pharmaceutical agents is critical since there is currently no radical treatment available, coupled with the anticipated surge in the number of patients afflicted with AD. Considering the outline as mentioned earlier, further research would undoubtedly prove beneficial in reducing both the prevalence and mortality rate of AD.

## Figures and Tables

**Table 2:** Diseases and their mechanisms associated with the onset and progression of AD.

Disease	Mechanisms Related to the Onset and Progression of AD	Reference
Hypertension	Increase white matter hyperintensities burden Modulations of the thickness of the entorhinal cortex (EC)	[59]
Obesity	Increase dysfunction in the dorsal hippocampus-dependent memory Dysregulation of the dorsal hippocampus (dHC) mitochondrial and lipid metabolism Disrupts noradrenergic transmission, neuronal function, and vascular integrity Angiogenesis Metal ion binding Apoptosis	[60]
Depression	Impacts cognitive domains, including attention, executive function, and psychomotor processing May affect episodic, working, and semantic memory Hippocampal volume reduction, impaired neurogenesis, and increased neuronal apoptosis	[61,62]
Diabetes mellitus	Nucleus accumbens atrophy Decreases gray matter volume and sulcal depth	[63]

**Table 3:** Antibiotics and their effects on behaviors and cognitive functions.

Antibiotic	Species	Effects	Reference
Antibiotic Mixtures (Ampicillin, Streptomycin, and Clindamycin)	Mice	elevated anxiety-like behaviors elevated depression-like behavior spatial memory impairment	[202]
Amoxicillin/Clavulanic acid	Rats	improvement in memory, decrease in apoptosis	[203]
Rifampicin	Mice	inhibition of Aβ oligomerization and microglial activation improvement in memory and decrease in tau hyperphosphorylation	[204]
Rapamycin	Mice	improvement in spatial learning and spatial memory reduction of Aβ deposits increase in nitric oxide production	[205]
Doxycycline	Mice	improvement in memory without decreasing plaques anti-inflammatory effect by decreasing microglial activation	[201]
Minocycline	Rats	improvement in memory and spatial learning interleukin-1β and tumor necrosis factor-alpha reduction in the hippocampus antioxidant effect	[206]
